# Accuracy of Using Generative Adversarial Networks for Glaucoma Detection: Systematic Review and Bibliometric Analysis

**DOI:** 10.2196/27414

**Published:** 2021-09-21

**Authors:** Ali Q Saeed, Siti Norul Huda Sheikh Abdullah, Jemaima Che-Hamzah, Ahmad Tarmizi Abdul Ghani

**Affiliations:** 1 Center for Cyber Security, Faculty of Information Science & Technology Universiti Kebangsaan Malaysia Selangor Malaysia; 2 Computer Center Northern Technical University Ninevah Iraq; 3 Department of Ophthalmology Faculty of Medicine Universiti Kebangsaan Malaysia Cheras, Kuala Lumpur Malaysia

**Keywords:** glaucoma, generative adversarial network, deep learning, systematic literature review, retinal disease, blood vessels, optic disc

## Abstract

**Background:**

Glaucoma leads to irreversible blindness. Globally, it is the second most common retinal disease that leads to blindness, slightly less common than cataracts. Therefore, there is a great need to avoid the silent growth of this disease using recently developed generative adversarial networks (GANs).

**Objective:**

This paper aims to introduce a GAN technology for the diagnosis of eye disorders, particularly glaucoma. This paper illustrates deep adversarial learning as a potential diagnostic tool and the challenges involved in its implementation. This study describes and analyzes many of the pitfalls and problems that researchers will need to overcome to implement this kind of technology.

**Methods:**

To organize this review comprehensively, articles and reviews were collected using the following keywords: (“Glaucoma,” “optic disc,” “blood vessels”) and (“receptive field,” “loss function,” “GAN,” “Generative Adversarial Network,” “Deep learning,” “CNN,” “convolutional neural network” OR encoder). The records were identified from 5 highly reputed databases: IEEE Xplore, Web of Science, Scopus, ScienceDirect, and PubMed. These libraries broadly cover the technical and medical literature. Publications within the last 5 years, specifically 2015-2020, were included because the target GAN technique was invented only in 2014 and the publishing date of the collected papers was not earlier than 2016. Duplicate records were removed, and irrelevant titles and abstracts were excluded. In addition, we excluded papers that used optical coherence tomography and visual field images, except for those with 2D images. A large-scale systematic analysis was performed, and then a summarized taxonomy was generated. Furthermore, the results of the collected articles were summarized and a visual representation of the results was presented on a T-shaped matrix diagram. This study was conducted between March 2020 and November 2020.

**Results:**

We found 59 articles after conducting a comprehensive survey of the literature. Among the 59 articles, 30 present actual attempts to synthesize images and provide accurate segmentation/classification using single/multiple landmarks or share certain experiences. The other 29 articles discuss the recent advances in GANs, do practical experiments, and contain analytical studies of retinal disease.

**Conclusions:**

Recent deep learning techniques, namely GANs, have shown encouraging performance in retinal disease detection. Although this methodology involves an extensive computing budget and optimization process, it saturates the greedy nature of deep learning techniques by synthesizing images and solves major medical issues. This paper contributes to this research field by offering a thorough analysis of existing works, highlighting current limitations, and suggesting alternatives to support other researchers and participants in further improving and strengthening future work. Finally, new directions for this research have been identified.

## Introduction

### Medical and Statistical Overview

Blindness and visual impairments often result from cataracts, age-related macular degeneration, and glaucoma [1,2]. Glaucoma is a neurodegenerative disease that damages the optic nerve and causes visual field loss [3]. As it is an asymptomatic disease, it is known as the silent thief of sight [4], and patients are unaware of the infection until their vision is irreversibly impaired. Among affected individuals, 50% are ignorant of the disorder [5-7]. Early phases of glaucoma have no symptoms or visual field changes [8]. As the disease progresses, a slow narrowing of the visual field can occur. If left untreated, glaucoma may contribute to total blindness [9]. Loss of vision usually begins on the eye’s side and then approaches the middle.

Statistically, glaucoma affects millions of people globally, with more than 64 million cases recorded in 2013, and other studies have estimated that 76 million people will be affected by 2020 and 111.5 million by 2040 [9,10]. Glaucoma is the second leading cause of blindness worldwide, preceded by cataracts [11], and it impacts 4.5 million individuals [9,12], more than 10% of the gross population [10]. Owing to the asymptotic function of glaucoma, approximately 70% of individuals with glaucoma are unaware of the illness’s existence [13,14] in the early stage. Thus, we need to provide an early detection and evaluation method [15]. Once glaucoma is detected, a more effective follow-up takes place as a cure can slow down the transmission of the disease [8].

**Figure 1:**
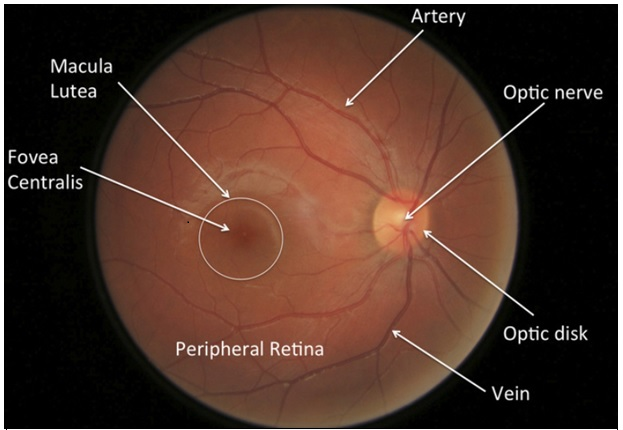
Fundus image structure.

Cataracts may be reversed by surgery, while glaucoma causes lifelong blindness. Elevated intraocular pressure (IOP) is the most common cause of glaucoma. The tonometer measures IOP. However, IOP is not always an accurate and adequate indicator of glaucoma, because glaucoma does not always cause a rise in IOP [16] but rather a deterioration of the optic nerve head (ONH). Visual information flows through the ONH to the brain. The ONH consists of a bright spherical area called the optic disc (OD) and a wider circle-like area called the optic cup (OC). Figure 1 shows these structures in ocular images.

ONH assessment is a widely used glaucoma screening tool that utilizes differential division to distinguish between glaucomatous and normal images [17]. Manual calculations of ONH geometric structures, such as the cup-to-disc ratio (CDR); inferior, superior, nasal, and temporal (ISNT) rule; disc diameter; and rim area, are recommended as diagnostic features for glaucoma screening [18-20]. Among them, the CDR is a reliable therapeutic feature for early glaucoma screening and diagnosis [21,22]. Each of the derived CDR parameters (diameter or area) is the ratio between the OC and the OD. CDR values rise when the illness progresses and become higher than approximately 0.6-0.7 when the patient has a stronger chance of developing glaucoma [23]. Based on an earlier study [24], a CDR of at least 0.65 is deemed glaucomatous in clinical practice. The CDR score tracks the development of glaucoma over time, effectively screening the condition early [25]. Currently, to check for retinal diseases, specialists tend to manually extract the blood vessel (BV), OD, or OC from retinal images. Accurate segmentation of the retinal structure is very important during the diagnostic process. However, doing this process manually is very labor intensive, time consuming, and risky in terms of human mistakes. Furthermore, the analysis results may lack objectivity, as different experts may produce different results. Therefore, it is important to automate retinal image segmentation/classification while minimizing expert interference.

### Research Background

The development of medical imaging technology has helped to accelerate the detection of diseases. Additionally, several studies have been conducted using image processing techniques to automatically process medical images without the intervention of experts [26]. Several studies [22,27,28] have examined vascular tracking and OD and OC segmentation using fundoscopic images. The main segmentation techniques depend on visual features such as color and contrast thresholding, region segmentation, and boundary recognition. Such methods use a learned classifier to classify pixels as foreground pixels (eg, OD, OC, or BV) or as background pixels (regions out of the area of interest) [29,30]. However, most of these methods are based on hand-crafted features (eg, texture, red green blue [RGB] color, gradient and Gabor filter), which are susceptible to low image contrast, pathological regions, and have a lack of deep feature extraction.

In recent years, automatic learning has been significantly improved with the assistance of machine learning (ML) techniques [31]. According to several studies [32,33], ML and deep learning (DL) algorithms have evolved to the point that they can compete with and sometimes even outperform humans on certain tasks, such as object detection [34] and image classification on ImageNet [35]. Currently, deep learning methods (DLMs) are an active research field because they can automatically generate and learn extremely complex features from input data. In particular, DLMs with deeper and complicated perceptron layers [eg, convolutional neural networks (CNNs)] have shown better performance in object detection than other methods [33]. Researchers have attempted to use various types of architectures, such as GoogLeNet [36], AlexNet [33], and DenseNet [37], for glaucoma diagnosis with the introduction of deep neural networks. Such research mainly focuses on 2 aspects: using DL for complex and deep feature extraction and utilizing medical features and spatial domain knowledge in the detection process. However, the use of deep fully connected networks is susceptible to imbalanced learning problems such as high false-negative or false-positive rates, leading to more fake or skinny branches than those of the ground truth [38,39]. In other words, retinal BV segmentation still has issues such as false pathological information segmentation and low microvascular segmentation [40].

For addressing complex learning issues, deep architectures often have advantages over shallow architectures; for example, deep CNNs have demonstrated significant efficiency improvements over conventional vision-based models [41]. A fully connected convolutional network has been used to address insufficient public data. Such methods, however, create very fuzzy vessels with false positives along with tiny and weak roots. This error primarily occurs because the CNNs used in current methods depend solely on pixel-level objective feature to equate the standard image to the image created by the model and are incapable of adapting actively to the fundus image of the natural vascular structure [42]. Empirical studies have proven that deep CNNs can learn invariant representations and attain human-level success if sufficient training data are provided. However, one of the leading shortfalls of DLMs is the lack of available data. Medical data annotation often requires specific domain experts. This shortage leads to the need for CNN training approaches with a limited number of annotated data. However, this can easily lead to underfitting, and as a result, high error rates on both training and testing data are recorded. Lahiri et al [43] demonstrated the effectiveness of using generative adversarial networks (GANs) [44] to perform some discriminative task with only 0.8%-1.6% of the amount of annotation data used by other methods.

GANs belong to the family of unsupervised learning algorithms that have proven their merits in generating synthetic images close to real images and solving image-to-image translation problems in the natural domain [45,46]. GANs have gradually shown their extraordinary ability and have started to shine brilliantly in various application fields [45,47,48]. Inspired by the prevailing learning capability of GANs, Wu et al [49] proposed the generative adversarial network with U-net, referred as (U-GAN), which includes an attention gate model in the generator and a densely connected convolutional network to segment the BVs automatically. Lahiri et al [50] proposed deep convolutional GANs (DCGANs) for retinal segmentation to segment the region of interest (ROI) from a given image. In addition to segmentation tasks, the synthesis of retinal images is a large part of the literature. Haoqi and Ogawara [51] trained a GAN model to learn the mappings of vessels from retinal images to segmented images for training a model to generate a synthesized image close to a given real image.

To date, several review articles summarizing the technology of DL in ophthalmology have been published [20,52-55]. Nevertheless, none of them have particularly focused on the emerging breakthrough GAN techniques using fundus photographs. Moreover, despite the rapid development of telecommunication technology, only a few study groups have examined the possibility of integrating artificial intelligence (AI) technologies with teleophthalmology [56]. To the best of our knowledge, no researchers have adopted telescreening for glaucoma using DL techniques, particularly the GAN.

Shedding light on the importance of telecommunication technology in DL techniques is a current and very urgent need. Alongside the emergence of newer low-cost handheld devices, glaucoma screening will become more available, even to distant and poor communities. In addition, maintaining social distance is very important for mitigating the spread of the coronavirus pandemic. This paper summarizes the work in the literature on glaucoma diagnosis and highlights the challenges and gaps of current studies to uncover the possibilities of filling these gaps with the recommended suggestions. We aim to elucidate all research efforts, such as the GAN architectures mentioned earlier, that have been developed in response to the new and disruptive technology, mapping the research landscape from the literature onto a coherent taxonomy of the key features that characterize such an emerging line of research. Finally, the future work of this research will be proposed and described in detail.

## Methods

### Basic Theory of GANs

**Figure 2:**
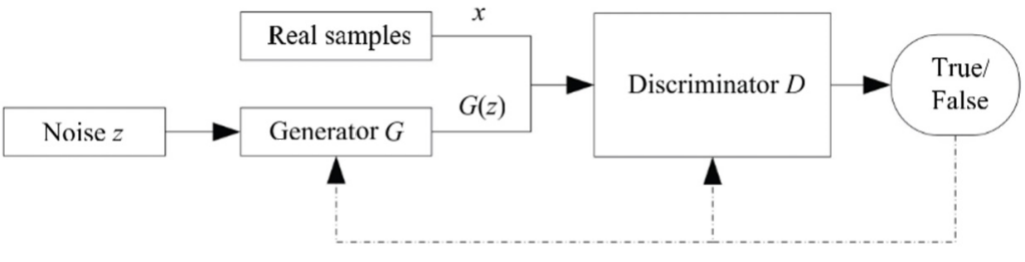
GAN architecture. GAN: generative adversarial network.

We start by reviewing the concept of GANs [44]. GANs consist of 2 separate neural networks, a generation network (G) and a discriminator network (D), plus a noise vector (z) sampled from a known distribution (eg, a Gaussian distribution), which is used to generate data points (fake samples; see Figure 2). A 2-player min-max game inspires the basic idea of this technique. The goal is to train the generator G to learn to capture the potential distribution in the real data sample and generate a new sample close to the real data to deceive the discriminator. The discriminator D is a binary classifier that attempts to discriminate whether the input data are real or fake [44]. To win the game, both G and D need to continuously improve their generation and discrimination capabilities, respectively. The training process lasts until both G and D reach a convergence point (Nash equilibrium), where G generates an output distribution very close to the real data distribution [42,57,58].

Mathematically, let G be parameterized by θ, which takes random noise z as input and produces synthetic images G(z) as output. The generated G(z) is mapped from a distribution G(z; θ) ∼ *p_g_*. Additionally, the training data set x is sampled from the real data distribution *p*_data_, and the objective function of the generator network is used to train G to approximate *p*_data_ using *p*_g_. By contrast, the discriminator (D) takes either the original image x or G(z) as input and indicates whether the input is from a true data distribution (x) or a synthetic data distribution G(z) by outputting a probability of D(x) or D(G[z]). This can be seen in the followig equation, where *p*_data_(x) is the true data distribution and *p*_z_(z) is the noise distribution.



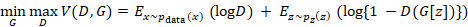



However, the training mechanism of such a model is critical. Unbalanced training between the G and D networks leads to model collapse. This happens when D is trained much better than G. In this case, D is able to easily discriminate between the real and synthetic images generated by G and reject all its outputs; thus, the loss log{1 − D(G[z])} saturates, and G learns nothing from the zero gradient. To avoid the model collapse issue, the loss function of G should be trained to maximize logD(G[z]) instead of minimizing log{1 − D(G[z])}. This can avoid the saturation of the gradient and provides the same gradient direction as that yielded by the old loss function.

### Extension Models of GANs

The first GAN [44] was composed of fully connected layers. Later, the DCGAN [59] introduced the use of fully CNNs to increase training stability and improve efficiency. Since then, many GAN models have followed this set up as the main components of GAN architecture. Unlike the DCGAN, the Wasserstein GAN (WGAN) [60] increases the permutation in the fully connected layer. In this model, the Wasserstein distance metric is used instead of the Jensen–Shannon divergence to measure the distance between the generated data distribution and the real data distribution. Therefore, the problems of model collapse and training instability were partially solved in this model. Subsequently, an improved version of the WGAN called the WGAN-GP (gradient penalty) [61] was proposed. The WGAN-GP depends on gradient penalty replacement so that it can solve slow training problems encountered by the WGAN. Moreover, inspired by the WGAN, Mao et al [62] proposed the least-squares GAN (LSGAN) to improve the quality of the generated images. The main idea of the LSGAN is to use a new loss function in the D network for smooth and unsaturated gradients.

The original GAN randomly generated a date distribution that is beyond our control, as the output depends on random noise. Therefore, a conditional GAN (cGAN) was invented to add a vector c as a conditional input to the noise vector z so that the generator could generate the required data. Hence, the generator output of the cGAN was defined by G(c,z).

Since the cGAN was proposed, many articles have used the cGAN applications, for example, Pix2Pix [45], a cGAN-based technique proposed by PatchGAN to map a set of images to another image using N × N pixels. It classifies each N × N path of the image and averages all the scores of patches to obtain the final score for the image. The main limitation of Pix2Pix is that it requires images x1 and y1 that are paired with each other in the training stage. By contrast, CycleGAN [47], which is also a cGAN-based technique, utilizes an image translation method that does not need paired data, even though Pix2Pix still outperforms CycleGAN’s remarkable margin.

Another variation of the GAN combines a variational autoencoder (VAE) and a GAN in a single model named VAE-GAN [63]. The idea behind this technique is to exploit the strength of both the GAN and VAE, as the GAN can generate sharp images but misses some modes while the VAE produces blurry images but with a large variety. Studies have demonstrated that VAE-GAN images are better than those produced by the VAE or GAN alone.

### Information Sources

Guided by [64], we conducted a comprehensive search to find all GANs-based articles related to glaucoma by searching the best and most reliable libraries: (1) Scopus, (2) ScienceDirect, (3) IEEE Xplore, (4) Web of Science, and (5) PubMed Central. This collection includes technical and medical literature, perfectly reflecting all research activities in this discipline.

### Study Selection Procedure

The method for choosing appropriate studies was on the basis of 2 stages: screening and filtering. Successively, both stages met the same criterion for inclusion and exclusion. Both duplicates and unrelated studies by title and abstract skimming were omitted during the first stage. Then, the result in a set of papers was entirely read, analyzed, and summarized in the filtration stage.

### Search

This work was carried out between March 2020 and November 2020. Various keyword combinations were used in the search of highly reputable libraries (IEEE Xplore, Science Direct, PubMed, Scopus, and Web of Science). Our search query consist of 2 parts that are connected with each other using the operator “and.” The following set of keywords (“glaucoma,” “optic disk,” “blood vessels”) and (“receptive field,” “loss function,” “GAN,” “generative adversarial network,” “deep learning,” “convolutional neural network,” “CNN,” Encoder) belong to the first and second parts, respectively. The operator “or” is used to connect keywords within the same part. Based on this, our study scope is formulated.

**Figure 3:**
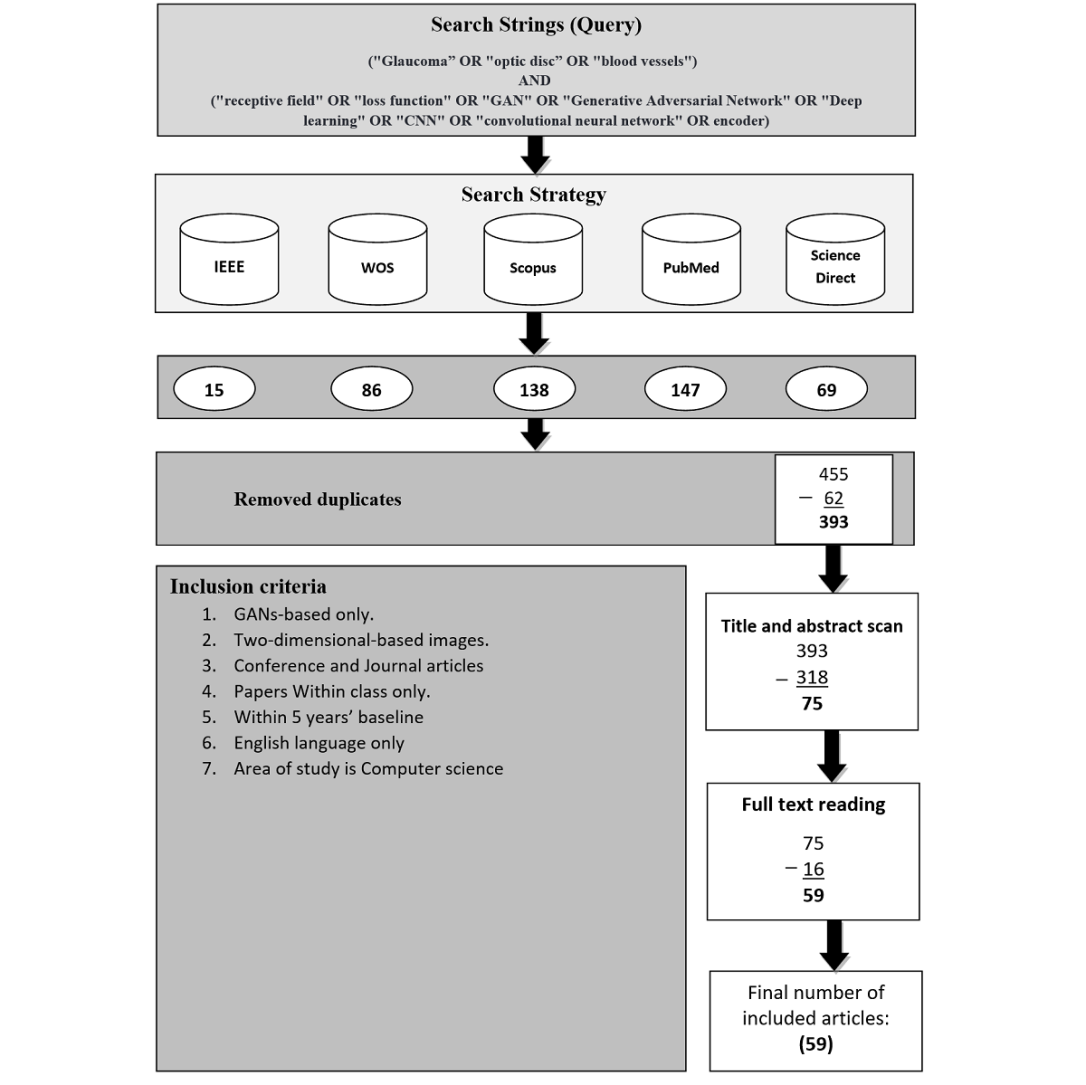
Flowchart of the study selection with the research query and inclusion criteria. GAN: generative adversarial network; WOS: Web of Science.

The quest focused on different journals and conferences and omitted books and all other forms of literature. Therefore, we mainly concentrated on up-to-date and applicable scientific studies related to the use of GANs in retinal disease, especially glaucoma. Figure 3 shows the research query and inclusion criteria used in this work.

### Validity of the Collected Papers (Scope Validation)

The total number of keywords in the collected papers was 115. To validate our research scope, we analyzed these keywords and categorized them according to their co-occurrences. Then, we set a threshold indicating the co-occurrences of each keyword across all papers. Let k≥3, where k is a threshold. As a result, we obtained 15 keywords out of 115 that met the threshold. That is, each of these 15 keywords occurred at least three times in all the collected papers.

**Figure 4:**
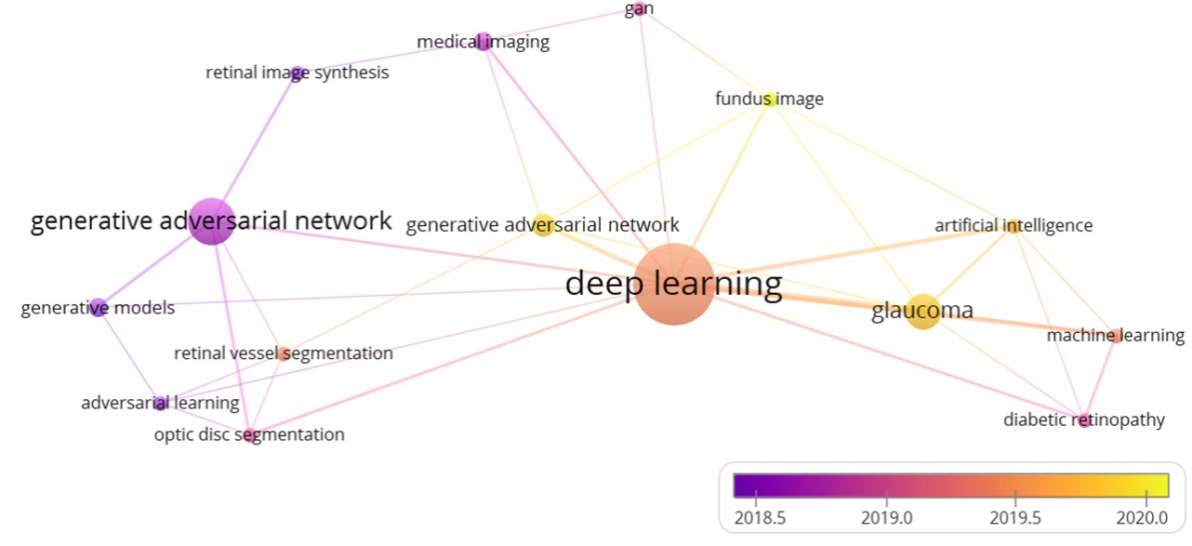
Scope validation diagram.

Figure 4 illustrates the connections of these 15 keywords to each other. The size of each circle indicates how frequently a single corresponding keyword occurred. The more frequently a keyword occurred, the larger circle size it gets, for example, the keyword “deep learning” has the biggest circle size in the diagram, which means it is the most frequently appeared keyword in the collected papers. The second factor is the color, which indicates how often a single keyword occurred per year. The last factor is the total link strength, which indicates the total connection of a keyword to other keywords. The more frequently 2 keywords appeared in the same article, the thicker is the line drawn between them. For example, the keywords “deep learning” and “glaucoma” were linked by a thicker line than the line between the keywords “generative adversarial network” and “glaucoma,” which means that both “deep learning” and “glaucoma” appeared together in the collected articles more than the keywords “generative adversarial network” and “glaucoma” did. This indication reveals that GANs have been used less than other DL techniques in glaucoma detection.

Table 1 shows the occurrences and the total link strength of the 15 keywords that met our threshold (k≥3). Deep learning is the most frequently occurring keyword, which has uniquely appeared 20 times in the collected articles and 27 times with other different keywords. GANs occurred 17 times, with 18 connections to other keywords, while the keyword glaucoma occurred 8 times, with 12 connections to other keywords. In conclusion, these highest scores for the aforementioned keywords empirically demonstrated the validity of our search query that is used to collect literature publications. Furthermore, it proves that our research scope revolves around 3 main keywords, namely, deep learning, generative adversarial network, and glaucoma, as they have the biggest circle sizes with the thickest connection among them.

**Table 1:** Keywords occurrence.

Keywords		Occurrences	Total link strength
**Techniques**			
	Deep learning	20	27
	Generative adversarial network(s)/GAN	17	18
	Artificial intelligence	3	8
	Machine learning	3	8
**Diseases**			
	Glaucoma	8	12
	Diabetic retinopathy	3	6
**Imaging**			
	Fundus image	3	6
	Medical imaging	4	5
**Papers’ contribution**			
	Adversarial learning	3	4
	Optic disc segmentation	3	6
	Retinal vessel segmentation	3	4
	Generative models	3	3
	Retinal image synthesis	3	3

### Inclusion and Exclusion Criteria

In this section, papers that met the criteria in Figure 3 were included. We taxonomized the included papers on a general and in-depth diagram consisting of 2 paper groups, namely, the development studies group and the reviews and surveys group. The papers in the first group were classified according to 8 consecutive layers. In the literature, researchers classified GANs into 2-4 categories; these categories were separately used by different researchers, as referenced accordingly in the points below. However, in our taxonomy, we combined them all. Furthermore, we added 4 more classification criteria as follows: (1) method architecture (direct, hierarchical, iterative) [32]; (2) model structure (2 players, multiple players) [65]; (3) GAN category (optimization function, structure, and conditional) [66-68]; and (4) generator backbone (U-Net based or CNN based) [69].

Further, we added 4 additional categories as follows: (1) type of GAN used in a paper (eg, variational autoencoder with GAN [VAEGAN], DCGAN, cGAN, CycleGAN); (2) discriminator’s receptive field (PixelGAN, PatchGAN, ImageGAN); (3) landmarks used during the segmentation/classification process (single, multiple); (4) paper contributions (segmentation, classification, image synthesis, mixed).

The exclusion criteria followed in this paper were as follows: (1) ML approaches, (2) 3D-based imaging methods (optical coherence tomography), (3) between-class papers, and (4) out-of-scope papers.

### Data Collection Process

All papers from different sources were summarized and saved in a single spreadsheet file for simplicity and a quick review. Significant remarks and comments were illustrated by full-text reading in our analysis scope and classification stage, which further refined our taxonomy. Finally, our results were summarized on an Excel sheet (Microsoft) and listed in a tabular format. The additional data set includes a list of articles, publishing source, articles’ abstracts and contributions, the tools used in papers, audiences, objectives, architecture-based categorization table, and a list of relevant figures.

## Results

### Overview

The cumulative number of articles in the original search process was 455. Eighty percent (364/455) of the findings released in 2018-2021 and 20% (91/455) in 2015-2017 were distributed as follows: 15 papers from IEEE Xplore, 86 from Web of Science, 138 from Scopus, 147 from PubMed, and 69 from ScienceDirect. Approximately 62 papers were duplicates across the 5 databases.

Later, 318 papers (not GAN based) were omitted after skimming through the articles’ titles and abstracts, leaving only 75 papers. Further screening via full-text reading was carried out on these 75 papers, which resulted in excluding 16 nonrelevant papers. A comprehensive reading was performed on the final 59 papers to create a general map to study this newly emerging methodology.

**Figure 5:**
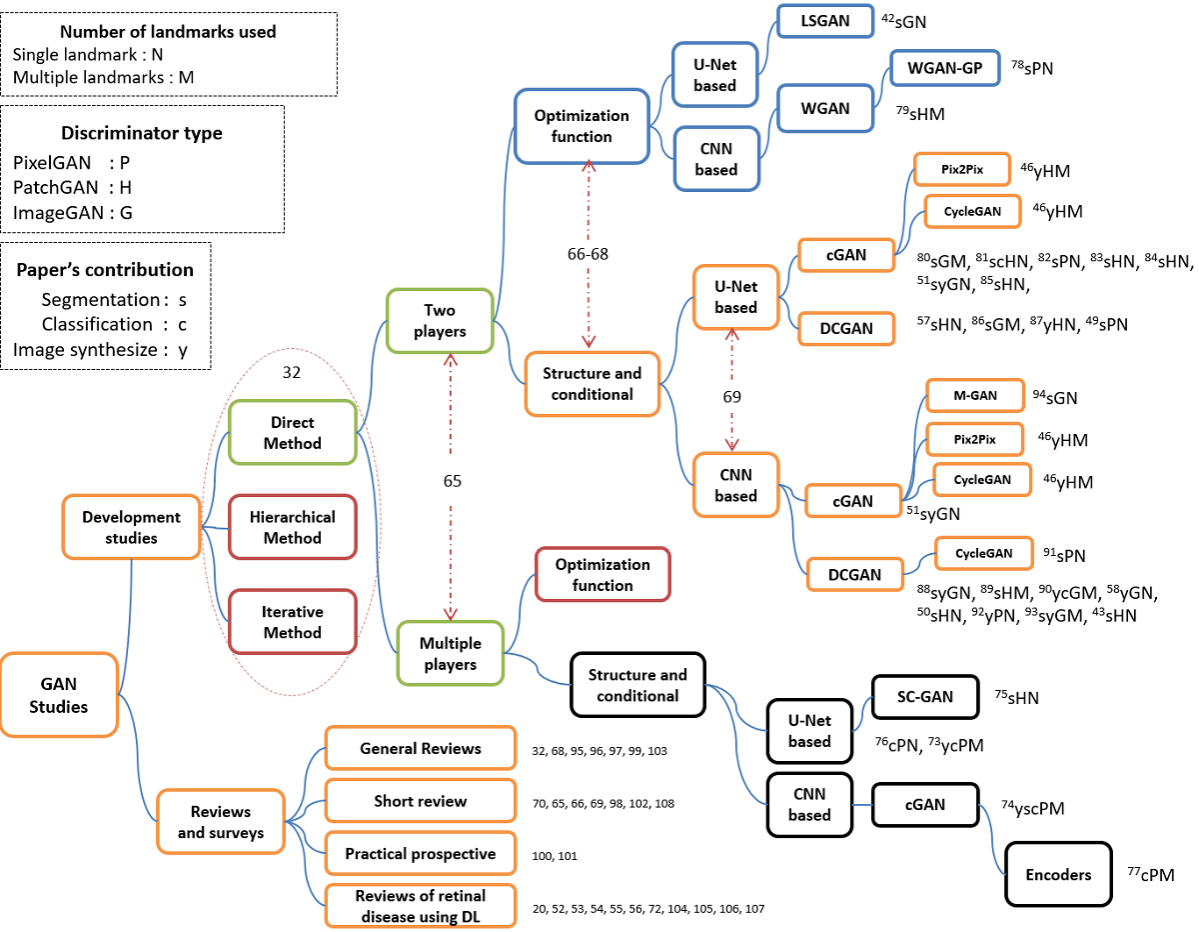
Taxonomy of the literature on glaucoma screening based GANs technique. cGAN: conditional GAN; CNN: convolutional neural network; DCGAN: deep convolutional generative adversarial network; DL: deep learning; GAN: generative adversarial network; LSGAN: least-square GAN; WGAN-GP: Wasserstein GAN-gradient penalty.

Of these 59 papers, 51% (n=30) focused on the development and training of various GAN models and real attempts to improve the efficiency of the network architecture to improve segmentation/classification precision, especially at an early stage of the disease with fewer false positives/negatives. Nearly 49% (29/59) of publications included general reviews and surveys relating to GAN technique and its variants; recent GAN applications, limitations, and potential future prospects; reviews of retinal diseases; various DL detection methods; general analytical knowledge such as the most frequently used data sets; and the countries contributing to the current research area. From all these observations, we got a thorough view on the literature, determined the general categories of the study scope, and boosted the taxonomic classification of the literature. Figure 5 presents the groupings of the GAN-based approaches used in the literature according to their structures or optimization functions.

Kumar and Dhawan [70] classified GANs based on their architectures or the loss functions used to train their generators. It is worth noting that the first 4 layers of our taxonomy have been separately used in other papers; therefore, inspired by those studies, we used these categories together as a baseline for our taxonomy. We added other categories to classify brief literature works in depth according to (1) their level of feature discrimination (PixelGAN, PatchGAN, or ImageGAN), (2) the numbers of landmarks used in the segmentation or classification process (a single landmark or multiple landmarks), (3) the backbones of the GANs used in the articles (eg, DCGAN [59], Info-GAN [71], WGAN [60], CGAN [45], Pix2Pix [45], and Cycle-GAN [47]), and (4) the contribution of each paper (eg, segmentation [s], classification [c], or synthesis [y]). In the following sections, we describe each category and provide some accompanying statistics.

### Development Studies Category

GANs were first developed by Goodfellow et al [44] in 2014. Although researchers have continuously attempted to improve the performance of GANs in various ways, such as weight regularization, new loss functions, weight pruning, and Nash equilibrium, it is still a new research field among deep learning techniques [70,72]. Only recently did this technique start to be adopted by researchers in the field of retinal disease, particularly glaucoma (roughly at the beginning of 2018). Therefore, the total set of papers that described various experiments and tools used for the detection or segmentation of retinal images included 30/59 (51%) articles.

Among these categories, it is notable in Figure 5 that the first 4 layers classified articles based on the method used (direct, hierarchical, or iterative) [32], the model structure [65], the architecture category (optimization function or structure and conditional based) [66-68], and the generator’s backbone (CNN based or U-Net based) [69] consecutively.

In the first layer, all the literature work followed the direct methods. This means that all these methods follow the philosophy of using 1 generator and 1 discriminator, and the structures of the G and D are straightforward without any branches. None of the articles used hierarchal or iterative methods; this reveals a new opportunity to apply GANs in the field of retinal disease.

The second layer classified articles based on the number of players. Nearly 25/30 (83%) articles used 2 players, and only 5/30 (17%) articles utilized multiple players. In the latter case, some studies used 3 player-based methods [73-75], with the frameworks of [74] and [75] comprising segmentation, generator, and discriminator networks. In the study by Liu et al [74], the segmentation network and generator enlarged the training data set to improve the segmentation performance, while the discriminator solely focused on identifying fake image–label pairs to ensure compatible utilities. However, in Yu et al [75], the same architecture was used to synthesize images after performing traditional annotation-free methods to obtain coarse segmentations.

A slight difference was observed in Wang et al [73], where a pathology-aware visualization network was used instead of the segmentation network, with both pathology-aware visualization and the generator used to enhance the synthesized glaucoma images in specific pathological areas. The synthesized image was re-enforced to provide a heatmap close to that of the input reference image. The Patho-GAN can thus generate images of glaucoma fundus with clearer pathologies. In Yang et al [76], the VGG19 network was incorporated with the 3 players to find the topology structure loss, which was combined with the other 3 losses (adversarial loss, weighted cross-entropy loss, and total variation loss) to be used by the generator. However, in [77], the authors used 2 encoders, namely, E_s_ and E_t_, where (s) is the source domain and (t) is the target domain; these encoders were trained to impede the classification performance of the discriminators (D+, D–). In turn, D+ and D– were trained to distinguish between positive/negative source images and positive/negative target images, and finally, a classifier (C) tried to classify source/target images.

Following [66-68], we added a third layer to our taxonomy to classify papers as either structure-based or optimization-based methods. The majority of studies (27/30, 90%) at this level were structure- and conditional-based methods, while only 3/30 (10%) of the studies, namely, those in [42,78,79], were optimization-based methods with 2-player structures; none of these methods have been recorded as multiplayer-based structures.

Some researchers tend to use objective function–based methods by updating specific loss functions or using a combination of losses to overcome the model collapse of GANs. This occurs when the generator continuously generates images with the same distribution or generates images with the same texture themes or color as the original image but with marginal differences in human understanding [65]; for example, Ma et al [42] used a least-squares loss function instead of sigmoid cross-entropy. Therefore, their experiment greatly improved the segmentation accuracy of the utilized model on both the digital retinal image for vessels extraction (DRIVE) and structured analysis of the retina (STARE) data sets by forcing the generator to generate images with distributions close to those of the real images. In Tu et al [78], the authors used the WGAN-GP method to overcome the training instability of the traditional GAN and generate accurate probability maps of BVs. The WGAN-GP is an extension of the WGAN; it uses a gradient penalty instead of weight clipping to enforce the Lipschitz constraint. This type of GAN can be trained faster and generates higher-quality samples than those produced by WGANs [61,68,70,78]. Last, Kadambi et al [79] proposed a framework for domain adaptation guided by the Wasserstein distance metric instead of typical adversarial methods for more stable training and better convergence.

The subsequent layer in our taxonomy was to classify methods according to the generator’s backbone (eg, U-Net based or CNN based) [69]. Papers [42,46,49,51,57,73,75,76,80-87] represented about 50% of the studies (n=16) and were U-Net-based architectures. However, the other 50% of the papers [43,46,50,51,58,74,77-79,88-94] were CNN-based generators (n=16).

The study by Yu et al [46] was very intensive; the authors proposed multiple-channels-multiple-landmarks as a new preprocessing framework. They used a combination of landmarks (vessel trees, ODs, and OC images) to synthesize colored images with 2 types of GANs (Pix2Pix and CycleGAN). Additionally, they used a Pix2Pix architecture with 2 different generator structures (eg, U-Net-based and CNN-based). They empirically demonstrated that the Pix2Pix network with a ResU-Net generator using high-resolution paired images and multiple-channels-multiple-landmarks outperforms every single landmark-based GAN method regardless of their architectures. Furthermore, they were able to generate significant and realistic images.

**Figure 6:**
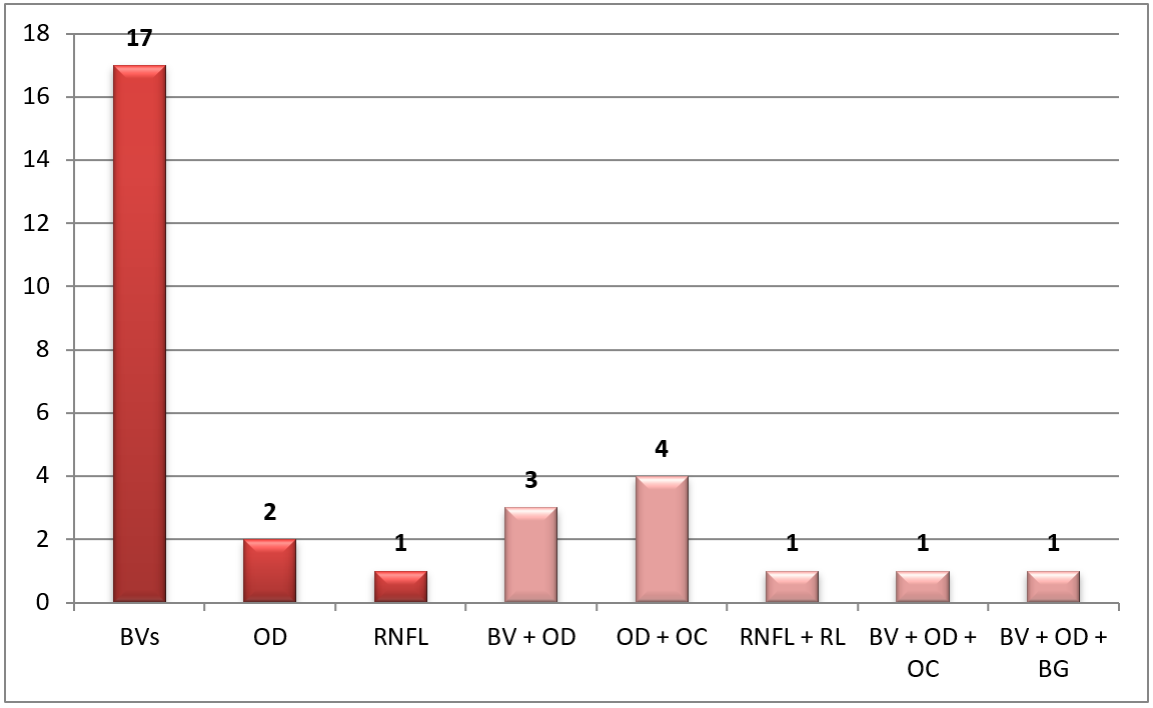
Distribution of papers per landmark(s). BG: background; BV, blood vessel; OC: optic cup; OD: optic disc; RL: rim loss; RNFL: retinal nerve fiber layer.

The next distinguishing level in our taxonomy addressed the landmarks used in the papers. As Figure 5 shows, references containing “N” letters refer to a single landmark (eg, the BV, OD, OC, retinal nerve fiber layer [RNFL], or rim loss [RL]). These references contributed to 20/30 (67%) of the total papers. Seventeen of them were BV-based methods [42,43,49-51,58,75,76,78,81-85,88,91,92,94]. Only 2 studies [57,81] were OD-based detection approaches, and 1 [82] utilized RNFL-based detection (Figure 6).

Another set of articles used multiple landmarks and was represented with an “M” letter in Figure 5. These articles contributed to 33% (10/30) of total papers. Some studies [80,90,93] used the BV and OD, while [74,77,79,86,89] used the OD and OC to classify the disease. In addition, Wang et al [73] used RL and RNFL, and Yu et al [46] used BV, OD, and OC.

The rest of the researchers used multiple landmarks, such as [74,77,79,86,89], which involved OD and OC segmentation. Studies [80,93] worked on BV and OD segmentation, and only Wang et al [73] used RNFL and RL. The rest of the papers used triple landmarks in their work, such as [58] and [90], which involved work on BV, OD, and background, and Yu et al [46] used BV, OD, and OC.

In the next layer of our taxonomy, articles were classified according to the discriminator’s receptive field. As illustrated in Figure 5, references with P, H, or G letters represent refer to PixelGAN, PatchGAN, or ImageGAN, respectively. ImageGAN papers were [42,51,58,80,86,88,90,93,94], while PixelGAN papers were [49,73,74,76-78,82,91,92]. In addition, PatchGAN papers were [43,46,50,57,75,79,81,83-85,87,89].

Isola et al [45] proposed a Pix2Pix-based conditional adversarial network (cGAN) as a general-purpose solution to image-to-image translation problems, and demonstrated that a 70 × 70 PatchGAN alleviates artifacts and achieves the best scores. Scaling beyond 70 × 70 to a full 286 × 286 ImageGAN did not appear to improve the quality of the results and, in fact, the latter model obtained a considerably lower fully connected network (FCN) score. This scaling mechanism may have been effective because there are more parameters in ImageGAN than PatchGAN and greater depth, which made it harder to train. By contrast, 3 studies [57,81,89] proved that the 64 × 64 Patch-SAN is the best, while one [84] concluded that a 120 × 120 patch is better than a 64 × 64 patch size. Studies [80,88] concluded that ImageGAN is better than PatchGAN. Last, pixel-level annotation [50] is much more tedious than image-level annotation.

Each reference in Figure 5 is denoted with a letter indicating the contribution of the relevant paper. Nearly 57% (17/30) of papers worked on the segmentation task and were denoted by (s), 17% (5/30) worked on image synthesis and were denoted by (y), and only 2 papers worked on the classification task and were denoted by (c). The remaining 6/30 (20%) papers worked on multiple tasks (eg, sc, sy, ysc). Multimedia Appendices 1-7 summarize the literature results reported in the papers.

### Reviews and Surveys Category

In this category, 2 sets of reviews were identified. In the first set, detailed discussion is presented about recent breakthrough techniques of GANs, their development, variations, and medical field applications. The second set shows the impact of deep learning on ophthalmology. In total, this category includes 29/59 (49%) papers.

For the first set, studies [32,65,66,68-70,95-98] provided detailed reviews about GANs including their basic background, theory, and implementations. Also, they present current research hotspots and proposed GANs in different applications. They provided the reader with a clear insight into GANs’ advantages and disadvantages, its different evaluation metrics, and proposed a bright prospect of this technique. Studies [32,95] focused on the importance of GANs, especially in medical field applications, and their capability to generate data through image synthesis technique without explicitly modeling the probability of density function. Wang et al [96] provided a further investigation of GAN in parallel intelligence. Another study, [99], discussed incorporating GANs in the signal processing community, showing different training methods, constructing GANs, and highlighting current challenges to their theories and applications. References [100,101] are practical prospective studies, and in [100], the authors tried to assess GAN algorithms and find the best architecture among all. However, they concluded that most of the models could achieve similar scores with enough hyperparameter optimization and random restarts. Additionally, they tried to overcome the limitation of evaluation metrics by computing precision and recall on several proposed data sets. Also, in [101], the authors reproduced the current state-of-the-art GANs, aiming to explore their landscape, discussing their pitfalls, and reproducibility issues. Turhan and Bilge [102] presented a comprehensive study about generative models such as GANs and autoencoders (AEs) and identified the relationship among them for better understanding and emphasizing on the importance of generative models. Oussidi and Elhassouny [103] proposed a starting point survey for those who have interests in deep generative models such as deep belief networks (DBNs), deep Boltzmann machine (DBM), restricted Boltzmann machines (RBMs), VAE, and GAN. They explained their building blocks, learning procedures, and limitations.

In the second set of articles, [52,54,72,104] presented an overview of DL applications in ophthalmic disorder using digital fundus images. They summarized the publicly available data sets used for different retinal diseases such as cataracts, retinopathy, glaucoma, and age-related macular degeneration. They also provided a detailed summary of the pros and cons of this emerging technique for both computer scientists and ophthalmologists and specified the clinical and technical aspects to address deep learning challenges and future directions. Some studies [56,105,106] discussed the importance of clinical considerations and potential challenges for clinical adoption and telemedicine integration to reduce cost, increase accuracy, and facilitate health care accessibility. Ting et al [53] described the importance of deploying deep learning algorithms within clinical settings. Hogarty et al [55] clarified the misunderstanding between ML and deep learning terms and presented an overview of AI and its development in the ophthalmology field. Mayro et al [107] also provided an overview of AI and deep learning DL applications in glaucoma detection using fundus images, optical coherence tomography, and visual field interpretation.

Other studies, [20,108], followed the systematic framework in their reviews: [20] discussed the main algorithms used for glaucoma detection using ML, indicating the importance of this technology from a medical aspect, especially retinal image processing, whereas [108] performed a systematic review on investigating and evaluating DL methods’ performance for automatically detecting glaucoma using fundus images.

**Figure 7:**
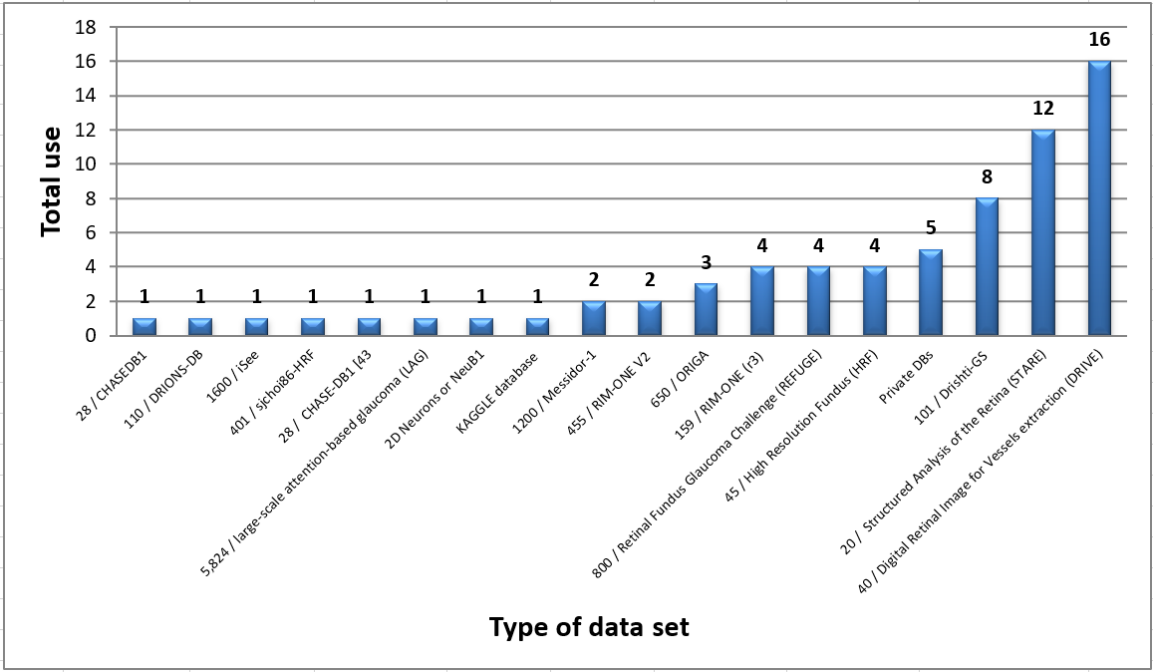
Total use of various datasets in glaucoma screening.

Figure 7 illustrates the publicly available data sets, their sizes, and how often researchers used them. Each data set is collected using a particular camera with different standards and used for a specific disease type. Thus, generalization is the key problem of DL approaches as described in the “Challenges” section.

As Figure 7 illustrates, DRIVE and STARE are the most frequently used data sets. In other words, researchers often rely on BV segmentation in the diagnosing process [72]. However, few researchers have used Messidor-1, high-resolution fundus, 2D Neurons(NeuB1), and CHASEDB. For OD and OC landmarks segmentation, DRIONS-DB, retinal fundus glaucoma challenge (REFUGE), ORIGA, RIM-ONE (r3/v2), and Drishti-GS were the most used, while seldom used is the large-scale attention-based glaucoma (LAG) data set, which is for RNFL and RL landmarks segmentation.

**Figure 8:**
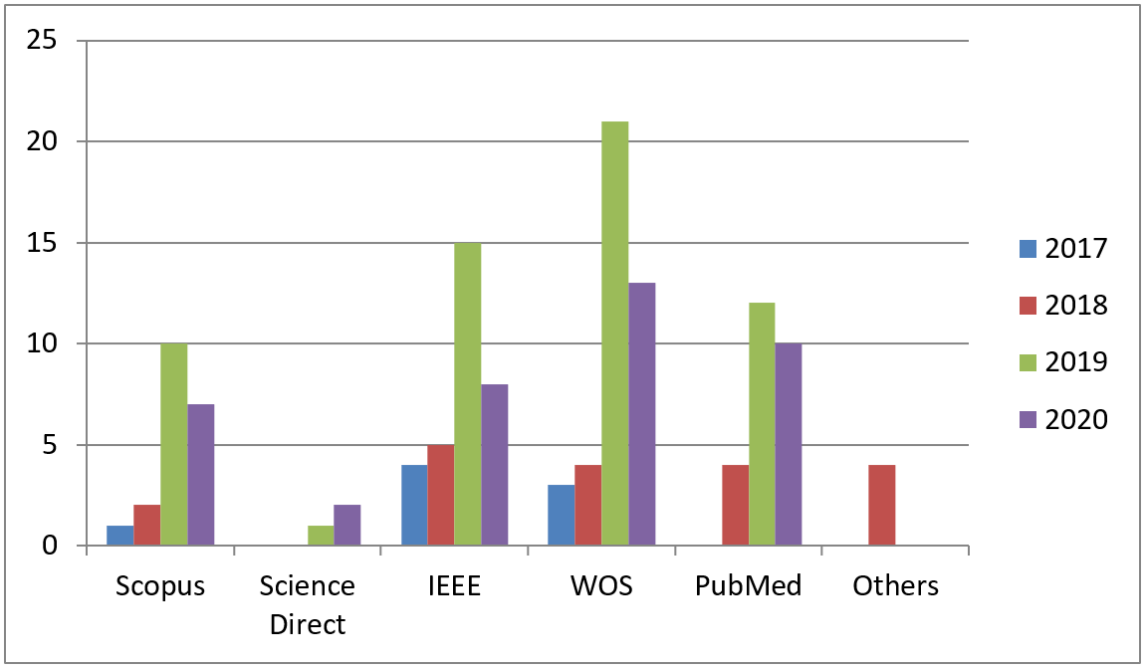
Distribution of papers per libraries. WOS: Web of Science.

Figure 8 shows the distribution of the collected papers per year regardless of their duplications. The statistics in Figure 8 indicates the recent interest of researchers to adopt GANs techniques. Furthermore, it reveals the need to explore this newly emerging technique in ophthalmology. Therefore, extensive further work is needed to cover this area of research.

This work has targeted 5 search engines: Scopus, ScienceDirect, Web of Science, IEEE, and PubMed, which are highly reputed and reliable resources for research. They include studies on implementation of deep learning techniques for different retinal disorder fields to help ophthalmologists and patients. Journal articles comprised 36 papers and only 23 were published in conferences.

**Figure 9:**
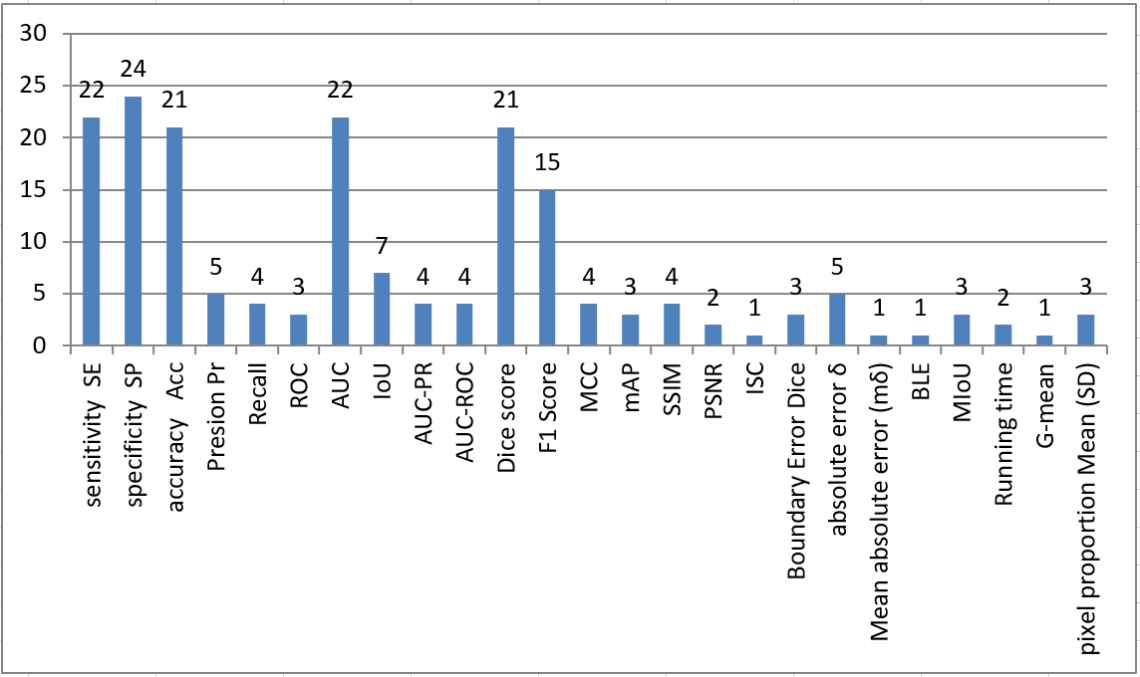
Distribution of frequently used evaluation metrics in glaucoma screening. AUC: area under the curve; BLE: Boundary Distance Localization Error; IoU: Intersection over Union; ISC: Image Structure Clustering; MCC: Matthews correlation coefficient; mAP: Mean Average Precision; MIoU: Mean Intersection over Union; PSNR: peak signal-to-noise ratio; ROC: receiver operating characteristic curve; SSIM: structural index similarity.

According to Multimedia Appendices 1-7, each paper has used a different set of evaluation metrics; thus, we concur with Yu et al [46] in concluding that there are no uniform evaluation indexes in the literature to evaluate synthetic and real images. To further clarify this issue, Figure 9 shows the distribution of evaluation metrics used in the collected papers. To present Multimedia Appendices 1-7 visually, a T-shaped matrix diagram in Multimedia Appendix 8 illustrates in the upper part, named “Metrics used,” the total use of each metric in all articles according to the used data set. Similarly, in the lower part, named “Task,” the diagram shows the total use of each data set in all articles according to a specific task (classification, segmentation, or synthesizing).

Based on the observations of the upper part of the diagram, the top 5 metrics (sensitivity, specificity, accuracy, area under the curve [AUC], F1 score, and Dice Co.) were used the most with various data sets. Furthermore, 87% (13/15) of metrics were mainly performed on STARE and DRIVE data sets, unlike other data sets, such as Rim-ONEv3 and Drishti-GS, that use another set of metrics (eg, F1 score, Dice Co, peak signal-to-noise ratio, structural index similarity, and δ) to evaluate the performance. This indicates the need to consider standard effect metrics in future research irrespective of the type of data set used.

By contrast, in the lower part, the segmentation task was reported as the most applied task in the collected articles, followed by images synthesizing, with the classification task being the seldom applied. Nevertheless, the best results reported were in a classification study by Bisneto et al [81], which utilized a combination of Dristh-GS and RIM-ONE data sets. They achieved 100% in sensitivity, specificity, accuracy, and AUC in OD/OC classification. Their method was based on cGANs with taxonomic diversity and distinction indexes. Although most of the studies are on segmentation tasks and professionally segmented BVs and ODs [42,78,80,91], they still lack segmenting fine and small vessels and suffer from false positives. By contrast, images synthesizing attracted increased interest of researchers, as it assists in overcoming the shortage of medical images. Some researchers, such as [58,87], have used GAN with adversarial AE to enhance the generated image and some others tend to rely on using different loss functions to better train G and D networks. However, generated images are blurry, noisy, and of low quality with lack of details. Other studies, such as [76,88,89,94], adopted preprocessing (eg, data augmentation, localization of ROI, automatic color equalization) and postprocessing (eg, Lanczos resampling method, morphological operation, contrast enhancement) to enhance the performance of their methods, and they experienced a further improvement in their segmentation result.

## Discussion

### Principal Findings

This study aimed to provide a detailed summary of the literature on retinal disease detection or segmentation, particularly glaucoma, using GANs and highlight the recent trends exhibited by researchers on this topic. We mainly focused on articles that worked on enhancing the segmentation or detection of the disease rather than improving GAN techniques. Furthermore, we provide a taxonomy of papers related to this area to further assist future research.

Several benefits may arise from our taxonomy. First, organizing tens of papers in a single diagram provides better understanding of literature work, as people with less experience may be confused if many papers remain unorganized. Second, the taxonomy helps sort literature works and activities into meaningful, easy to manage, and coherent frameworks. Third, it provides researchers with better insights into a given theme, thus finding current literature gaps and discovering new research directions. Last and most importantly, it helps highlighting articles’ strengths and weaknesses of a particular research scope.

From the developed taxonomy, we can quickly see that all the published papers followed the direct method of the GAN architecture; hence, there is an urgent need to discover the impact of the hierarchical or iterative method on glaucoma screening. Moreover, almost all of the researchers worked on BV segmentation, and very few used OD and OC segmentation, which are the most reliable indications of glaucoma according to ophthalmologists. Future GAN research should focus on disease classification rather than on the segmentation of retinal anatomy. Most of the literature studies faced difficulties in terms of the early detection of glaucoma and low segmentation of fine vessels; therefore, alternatives should be developed, for example, using the RNFL to indicate the early presence of the disease or exploiting the prior knowledge of vascular connectivity to improve upon the segmentation performance of the current methods. Although the RNFL is a good sign for early glaucoma screening and has been incorporated as one of the gold standards of glaucoma evaluation [109], very few studies utilized the RNFL with GANs. OD/OC segmentation may lead to interference with pathological aspects such as large genetic OD sizes. Based on the reviewed papers, we noticed that only one article [1] has used RNFL for glaucoma screening. Although that study achieved impressive results, the authors used a private data set.

Most of the previous studies concentrated on the segmentation task. As much as 17/30 papers worked on retinal landmark segmentation [1-17], while only 2 papers worked on disease classification [18,19], and 5 papers worked on image synthesis to address the lack of medical images [20-24]. However, the rest of the papers (6/30) performed multiple tasks (eg, segmentation and classification, synthesis and segmentation) [25-30]. In conclusion, more than 50% (17/30) of the literature worked on segmentation task and few researchers have worked on classification and synthesizing retinal images. Therefore, future studies should take these statistics into considerations.

In the following sections, the included papers will be discussed in detail. We present comprehensive diagrams showing the factors that motivate researchers to carry out their work in this area, highlighting their encountered challenges, and summarizing significant recommendations for addressing their faults in future work.

### Challenges

#### Overview

**Figure 10:**
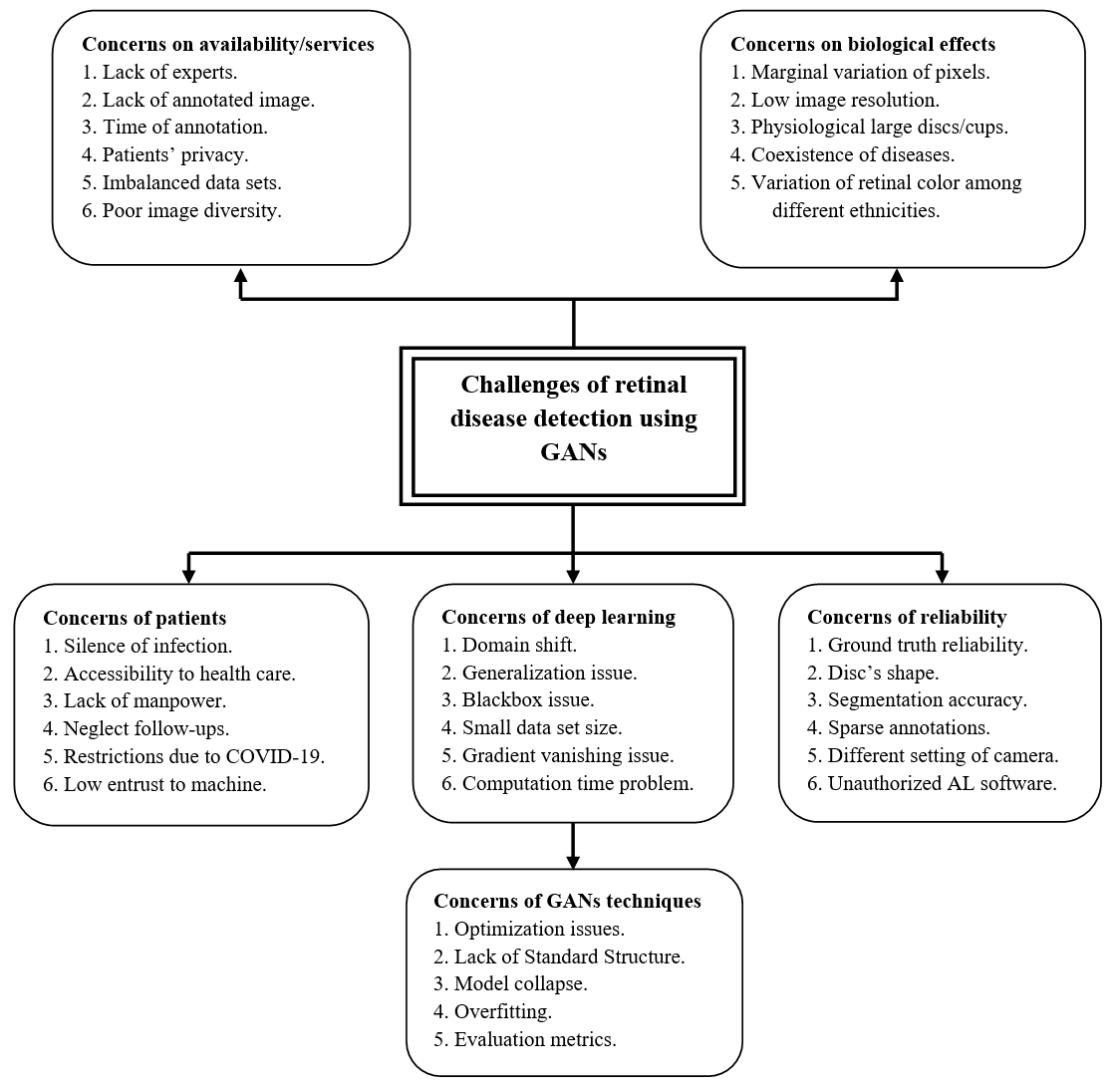
Challenges of glaucoma screening using GANs technique. AL: artificial learning; GAN: generative adversarial network.

Glaucoma is a serious disease. Therefore, researchers and developers attempt to exploit the magic of DL technique to help doctors and patients diagnose the disease at its early stage. However, various challenges hinder their expectations; some of those challenges implicitly exist in the nature of DLMs, or are somehow incorporated within DLMs (eg, data richness, diversity of data, and powerful hardware), besides the challenges of GANs architectures (eg, model collapse, optimization, Nash equilibrium, and evaluation metrics). All these challenges have been summarized and discussed in this section along with their relevant references to provide the readers with direct access to the original papers for further discussion. Figure 10 categorizes literature challenges into 6 groups to further assist discussion. Each group is indicated with a separate shape.

#### Challenges Related to Patients

The silent progress of glaucoma disease constitutes a crucial challenge worldwide. Half of the infected people do not experience any symptoms at early stages [5-7]. According to various studies, more than 60 million cases were diagnosed globally in 2013, and it is expected to exceed 75 million and 111 million cases by 2020 and 2040, respectively [9,10]. Especially among rural populations, China and India are considered to be the home to approximately 40% of glaucoma cases globally [110]. These populations, mostly in developing countries, suffer from difficulties in accessing medical centers, unavailability of experts, high costs of health care, and sustainability of health care services [111], in contrast to Western countries, where health care is cost-effective and different socioeconomic situations of patients are supported, and thus treatment for glaucoma remains affordable [106].

In addition, the recent pandemic, COVID-19, has enforced social distancing during communication. Therefore, there is a great need to promote ocular screening in conjunction with telemedicine as a remote monitoring tool [112], alongside the presence of handy cheap smartphones, whereby patients can collect their own IOP data themselves with accurate tonometers and free anesthesia [113]. Although DLMs positively affect both doctors and patients’ style in terms of decision making, cost affordability, and health care accessibility, there remain some serious challenges, such as technical and clinical challenges, interpretation of the results, and patient trust in machines [112]. Zapata et al [114] predict that very soon AI will start assisting specialists in achieving high levels of consistency and accuracy beyond human abilities.

#### Challenges Related to Reliability

Reliability is a key to adopting computer technology in the medical field. Deep learning techniques may misclassify segmenting some pixels due to low image contrast or heavy overlap between foreground and background pixels, leading to false-positive/false-negative result [57,81]. In some cases, doctors are dissatisfied with deep learning segmentation performance, as it is not as real as their expectations. Taking RNFL segmentation as an example, the segmentation results do not have specific geometrical shape of RNFLD as the gold standards and large segmentation errors of fundus images [83]. Furthermore, the variability of shape and extremely inhomogeneous OD structure appearance result in inaccurate CDR measurement compared with ideal ones [115-117]. In some cases, deep learning approaches neglect domain knowledge that doctors care about, such as CDR [118].

Existing methods often suffer from poor segmentation of the fine vessels [78,80] due to weak ability of antinoise interference or insufficient segmentation of vessels [49]; therefore, prior knowledge of BVs connectivity may improve the segmentation performance. Meanwhile, the low reliability of manual detection and the small size of public data sets increase the complexity of morphological assessment of nonglaucomatous optic neuropathy [119,120]. Robust ground truth labeling must be generated after a comprehensive evaluation, including structural imaging, clinical examination, and perimetry [121]. Doctors mostly decide the disease status. Although all clinical symptoms occur, it can lead to differences within annotators, and thus exaggerated annotations [52,106,122,123]. The reliability of glaucoma algorithms is restricted due to the lack of reference ground reality for glaucoma [115,124]. DLMs have a remarkable ability to address glaucoma. However, it is critical to have gold-standard algorithms for assessing and detecting glaucoma [54], as well as for editing or synthesizing images using the GAN techniques [97].

Sometimes, researchers tend to exclude low-quality or sparsely annotated images during the training phase; this kind of regime weakens the algorithm and leads to less reliability in real-life cases [111]. Furthermore, incorporating nonspecialists for image grading limits the reliability of identification [125]. Finally, although most of the reviewed papers have shown outstanding diagnostic performance, at times researchers do not mention some hyperparameter values used in the training stage, particularly when they use their own private dataset [112]. Excessive screening can result in overdiagnosis. DLMs could also be harmful if the diagnostic software is issued directly to patients, as future opportunities and risk of AI could be magnified [55].

#### Challenges Related to Biological Effects

Pathological change and image quality play a major role in the accuracy of glaucoma diagnosis [57,73,123]. Early and moderate glaucoma stages are considered one of the biggest challenges faced by ophthalmological practice due to the marginal variation size of CDR compared with normal eye [126]. Serener and Serte [127] have used ResNet-50 and GoogLeNet with transfer learning for early and advanced glaucoma detection, and found that GoogLeNet outperforms ResNet-50 with a trade-off performance between sensitivity and specificity. Besides, Bisneto et al [81] proposed GAN-based OD segmentation allied with an index of taxonomic diversity for extracting texture attributes aiming to detect early stages of glaucoma. They achieved outstanding results reaching up to 100% for accuracy and 1 for the receiver-operating characteristic curve. The misclassification of glaucoma and nonglaucoma is usually due to heavy overlap and extremely bad contrast between ocular structure and the background, leading to unsatisfied segmentation performance due to OC’s undistinguishable boundaries [116]. Low-quality images (blurring and contrast) can result in unreliable model predictions. Furthermore, the lack of a clear OC border increases the misclassification rate [128].

There is a trade-off between image’s quality and computational parameters of the network [129]. Therefore, the need for DLMs to downsample images into lower resolution (ie, 224 × 224) to reduce the computation time leads to reducing image contrast, and hence deteriorating key diagnostic parts of ocular images and weakening the capability to recover contextual information [86]. By contrast, performance of DLMs varied among ethnicities, for example, the Saudi population’s performance is not the same as on Western populations. The differences among populations is due to the richness of melanocytes in the retinal pigmented epithelium of darkly skinned people compared with Whites [52]. Therefore, data sets used in glaucoma detection must follow specific standards to ensure heterogeneity and diversity of images.

Multiple eye disorders such as high myopia or pathologic are another major challenge leading to false-negative and false-positive results [54]. The main reason for the incorrect segmentation of glaucoma in myopia cases is the alteration of the macula and optic nerve appearance. In addition, the use of RNFL imaging for glaucoma diagnosis in patients with diabetes should be made carefully [130]. Myopia affects macular and RNFL thickness measurements due to the thinning and stretching of these layers caused by the increased axial length and optical projection artifact of the scanning region [131]. Myopia mostly causes misclassification of glaucoma due to its irregular ONH appearance [132]. In severe myopic cases, the color contrast between the foreground (OC) and the neuroretinal rim decreases due to an increased pallor in the rim. Furthermore, the increased pixels’ values brighten the underlying peripapillary tissue and lead to difficult evaluation of the RNFL in the peripapillary area. In addition, torsion or tilting of the OD can occur, and the OD’s rotation can result in an oblique view of the ONH [128].

In other cases, it is hard to distinguish between physiologic large cups and glaucomatous cases because both cases share a common feature (eg, large CDR) [117]. Diseases such as OD edema, OD hemorrhage, and glaucoma frequently make segmentation of OD rather difficult [133]. By contrast, retinal BV segmentation also has inherent challenges such as incorrect segmentation of pathological details and low microvascular segmentation [40].

#### Challenges Related to Availability/Services

Time, efforts, and lack of experts are the main challenges of medical care centers [88,134]. Therefore, computers have been increasingly used for automatic retinal segmentation to serve as a second opinion to the doctors, improve the diagnostic accuracy, and reduce the tedious work of annotating images [43,46,135]. Particularly, GANs showed impressive performance in medical image synthesis and it is usually employed to tackle the shortage of annotated data or lack of experts [74,79,95]. Generally, medical images are usually rare, expensive, and full of patient privacy issues [51,88] and the publicly available data sets are often imbalanced in size and annotation [46,57,84]. In general, segmentation tasks suffer from an immense problem of class imbalance. Thus, the accuracy metric is not sufficient alone until concluding a system’s efficiency on both sensitivity and specificity. They should, however, be considered as an essential evaluation metric [72].

Diaz-Pinto et al [90] proposed a GAN method with semisupervised learning to develop a good image synthesizer to tackle the shortage of retinal image availability and support generalization ability. Additionally, Liu et al [136] created a large-scale glaucoma diagnostic fundus images (FIGD) database. They proposed the glaucoma diagnosis with a complicated neural networks method for automatic detection of glaucomatous optic neuropathy. Importantly, the method has the potential to be generalized throughout populations.

Various GAN-based methods have been proposed to mitigate image labeling [43,50,51,75,87,92]. However, this challenge remained open as the current literature results are still inaccurate (eg, fail to generate very thin vessels). Lahiri et al [43] concluded that the diversity of annotated images is more important than the actual number of annotations. Finally, rural areas experience difficulties in locating ophthalmologists. This also necessitates more future work to use telemedicine in ophthalmology [55].

#### Challenges Related to the Nature of Deep Learning

With the recent advancements in DLM methodologies, promising results in the field of ophthalmology have been obtained. Many GANs and CNNs models are proposed in computer vision. However, DL approaches face several difficulties, such as domain shift.

Domain shift is the disparity in appearance distribution between various data sets due to different camera settings, illumination variation, different screening angles, or out-of-focus ROI. As a result, domain shift hinders the generalization capability of deep networks [89]. In most literature, training and test data sets come from the same image distribution. However, this is not always the case in real life. Therefore, it may significantly damage the real-life applications if not handled beforehand [72]. Kadambi et al [79] proposed an unsupervised domain adaptation framework by allowing the model to learn domain-invariant features to enhance segmentation performance and generalization capability. Wang et al [77] tried to align the distributions of the source and target domains so that the labeled source images can be used to enhance the classification efficiency of the target domain.

Deep learning addressed many issues in the traditional methods of ML. However, it also brought new difficulties. The most crucial issue is the ambiguity of the diagnosing result; in other words, the blackbox problem [53,56]. DLMs are blackbox in nature and do not have diagnostic explanations to confirm their effectiveness in a real clinical setting. Wang et al [73] proposed a pathology-aware visualization approach for feature visualization using DNNs to explain better how decisions are taken by computer, and therefore find pathological evidence through computer-aided diagnosis. Furthermore, for this purpose, Zhao et al [115] proposed a weakly supervised model due to its ability to simultaneously learn the clinical evidence identification and perform the segmentation task from large-scale weak-label data that further improves glaucoma diagnosis.

The lack of publicly available data sets for training the model is another significant challenge concerning deep learning approaches. Therefore, Orlando et al [132] proposed a data set named REFUGE, which contains 1200 fundus photographs with standard gold segmentations and clinical glaucoma marks. Moreover, Li et al [137] created the LAG database containing 11,760 fundus photographs classified as either positive glaucoma (4,878) or negative glaucoma (6,882), which is the largest among the currently existing databases. According to Asiri et al [52], the key problem of constructing a robust deep CNN method is not the availability of broad data sets but instead the diversity of annotation of those images [43]. A major difficulty of each algorithm is its validity in multiple patient cohorts with diverse conditions. Therefore, for a DLM to be sturdy, it must be effective across various data sets [105].

Recent studies demonstrated that more complicated and informative image features might be discovered when growing the depth of the network [138,139]. However, as the network depth rises, deeper CNN has poor diagnostic efficiency due to the gradient disappearance issue or the gradient explosion problem [88,140,141]. Researchers mostly use shortcut links (skip connections) that skip one or more layers while training deep networks, as was the case with [88,126,128,129]. Alternatively, in GANs techniques, using WGAN or LSGAN gives a smoother gradient that contributes to stable training [42,79]. Another concern that should be considered before building up deep models is the *computation time*. As there is a trade-off between model’s depth and the efficiency, the deeper the architecture the greater the number of parameters it gets, which eventually increases computation time [140].

#### Challenges Related to GAN Technique

Despite all the ongoing developments and studies, GANs suffer from several challenges and weaknesses besides the challenges related to deep learning nature (eg, blackbox, generalization capability, computation time, and annotation cost). The most critical concern with GANs is the instability of the training process (Nash equilibrium point) [98,142]. Zhao et al [82] used the residual module that allowed easy optimization of competitive networks, while Tu et al [78] used WGAN-GP to alleviate training instability of the traditional GAN. Biswas et al [92] carefully adjusted hyperparameters to balance between the 2 networks (G and D). Park et al [94] improved learning performance and mitigated imbalanced learning by introducing new loss functions for the generator and re-designing the discriminator’s network. However, it remains challenging to determine which algorithm works better than others or what modifications are critical to enhancing the results. Lucic et al [100] found that most models could achieve comparable scores with appropriate hyperparameter optimization and random restarts. According to Kurach et al [101], the nonsaturating loss over data sets, architectures, and hyperparameters is sufficiently stable.

Besides, in GANs, the possibility of mode failure/collapse persists while training the model. Model collapse occurs when data generated from GANs mostly concentrate on very narrower modes (partial collapse) or 1 single mode (complete collapse) [68,99]. By contrast, if the discriminator becomes very strong during training, the generator gradient gradually decreases and eventually disappears. As a result, the generator learns nothing. The imbalance between generator and discriminator networks contributes to overfitting. Many approaches have been proposed to tackle these challenges; for example, Radford et al [59] aimed to address instability training issues, and Kadambi et al [79] created a new adversarial domain adaptation architecture, led by Wasserstein for better stability and convergence.

The lack of standard evaluation metrics is another big issue in GANs compared with other generative models. Inception score (IS), average log likelihood, Fréchet inception distance (FID), Wasserstein metric, etc. are quantitative measurements of GANs. There is no majority vote on which assessing measurement is the best. Different scores rely on various aspects of image generation. However, some measurements seem more plausible than others (eg, FID is more durable to noise). FID can compare the similarity between real and generated images [143], which is considered more effective than IS [70].

In conclusion, the main causes of GAN problems can be summarized as follows: (1) The distance calculation of the corresponding optimization (such as Kullback–Leibler divergence and Jensen–Shannon divergence) is unreasonable. (2) It is difficult to overlap the generated distribution with real distribution. Although the GAN technique is a new, interesting, and attractive field of study in many applications, further studies are needed to resolve the uniqueness of generated samples, poor convergence, and complete model collapse challenges.

### Motivations

**Figure 11:**
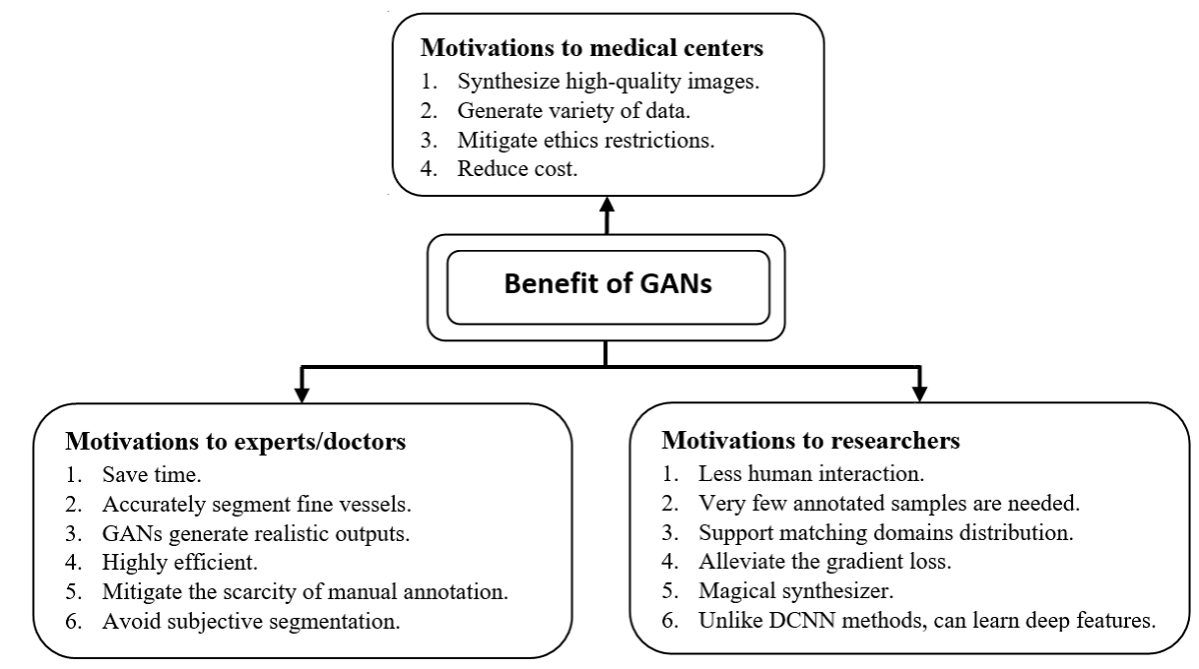
Benefits of GANs-based methods for glaucoma screening. DCNN: deep convolutional neural network; GAN: generative adversarial network.

Adopting deep GAN in ophthalmology is a promising and significant field of study. This section reports some of the literature’s characteristics, which we classified on the basis of references to support further discussion (Figure 11).

#### Motivations Related to Experts/Doctors

Detection of any retinal defects must be through analysis of ocular images. Analysis of retinal images, however, must involve trained physicians to analyze and assess digital color fundus images. Such a process requires a great deal of time and human work; therefore, GANs support doctors in mitigating this extensive bottleneck [50,51,91]. Furthermore, deep GANs techniques are unlike CNNs, where the same GAN approach could be applied to a wide variety of cases and still produce reasonable results [45]. GANs can detect the OD in fundus photos with pathological changes or irregular highlights [57,86]. In the case of vessel segmentation with CNN-based methods, outputs are usually blurry around small and weak branches or suffer from a problem of nonconnectivity of segmented vessels; however, GANs better segment capillary/thin vessels of fundus images [76,80,84], and thus serve as a second opinion to ophthalmologists [72]. GANs are the framework that allows to create and use practical outputs as a gold standard [44]. Therefore, these frameworks were adopted by Lu et al [83] due to their ability to generate the required specific geometry of RNFLD, which is close to ground truth with high precisions, accuracy, and fewer segmentation errors, despite the existence of multiple pieces of RNFL or low-contrast images. Thus, its segmentation results are much more trusted by doctors than CNN’s.

Adversarial learning avoids scarcity of manual annotation and subjective segmentation made by non-expert clinicians as this methodology is mainly data driven [72,85]. In glaucoma classification, enforcing GANs to synthesize images with similar visualization results as the reference image will help mitigate the drawbacks of binary labels (negative or positive) that limit the visualization methods to recognize pathological facts underlying diagnosis by DNNs [73].

#### Motivations Related to Researchers

Deep learning in retina images is very effective and useful [72]. However, they are often affected by domain shifts across data sets. As a result, a generalization of DLMs was severely hindered. Therefore, researchers tend to exploit generative adversarial learning for domain adaptation by encouraging the target domain predictions to be close to the source ones [79,89]. Domain adaptation is often used to overcome the lack of large pixel annotation using off-the-shelf annotated images from other relevant domains. Alternatively, researchers exploit the existence of a large amount of unlabeled data to train a classifier using the power of DCGAN in a semisupervised learning scenario [90]. Semisupervised learning is in the middle way between unsupervised and supervised learning; therefore, less human intervention is required when combined with GANs for better semantic segmentation [74]. Using GANs techniques, Lahiri et al [43] performed image segmentation with very few annotated samples (0.8%-1.6%), nearly 500-1000 annotations. Further, Zhao et al [93] proposed an image synthesizer using GANs with style transfer and then integrated the outputs into the training stage to boost segmentation efficiency using just 10 samples.

With deep adversarial learning, researchers aim to reduce domain discrepancy [144,145] by improving the quality of the generated outputs to be as close as possible as the inputs. Wang et al [77] exploited label information for matching domain distribution. Ma et al [42] applied the least-squares loss function instead of sigmoid cross-entropy to generate images with distribution close to the real ones and also alleviate gradient vanishing problems. Furthermore, Liu et al [57] added a patch-level adversarial network to enhance image consistency between ground truth and the generated samples, which further boosts segmentation performance.

GANs are capable of learning the mapping from the input image to the output image as well as learning a loss function to train this mapping [45], unlike existing DLMs, which use a unified loss function for retinal vessels segmentation, thereby producing blurry outputs with false positives around faint and tiny vessels [84], which is in contrast to GAN variations (eg, WGAN-GP and M-GAN) that provide accurate segmentation results around small and weak branches [78], reduce low microvascular segmentation [94], and preserve the connectivity of arteriovenous vessels [76]. Moreover, AEs and GANs in a single system facilitate generating vessel maps without the previous existence of retinal vessel tree [87]. Besides, unconditional GANs can synthesize retinal images without using prior vessel images [92].

Although researchers recommend using DCNN for efficient segmentation tasks [146], the existing limitations of DCNNs are insufficiency of feature extraction, weak generalization capability, and poor capability to recover low-context information, unlike GANs, which are used to alleviate these problems as in Jiang et al [86], who proposed GAN with transfer learning, data augmentation, and skip connection concepts to overcome these challenges. Bisneto et al [81] impressively improved glaucoma segmentation and classification results using GANs allied with texture attributes identified by taxonomic diversity indexes. They achieved promising results (sensitivity, specificity, and accuracy of up to 100%).

For optimizing network complexity, Wu et al [49] applied the attention Gates technique in a standard GAN to encourage the propagation of features, promote reuse of features, and greatly reduce network parameters when paired with DenseNet instead of conversion layer. Alternatively, using dilated convolutions in the generative networks effectively expands the generator’s receptive field without the number of calculations [82]. Adversarial training has been shown to improve the long-range spatial label interaction without expanding the segmentation network’s complexity [147].

#### Motivations Related to Medical Centers

We think the best medical treatment is achieved when the doctor–patient relationship is built on honesty and concern. DL cannot substitute real relationships, but can complement them [104]. GAN architectures are versatile. For various training samples, the objective feature can be re-designed and more free model designs can be used [98]. The extraordinary feature of GANs in the medical field is synthesizing high-quality images with global consistency(eg, color consistency and both BV and OD occupy the same proportional area as the real images) [58,92]. Bisneto et al [81] proposed a method that learns the mapping function between retinal landmarks (BV, OD, and OC) and synthesizes images using the 3 channels (RGB). Furthermore, the method exploits the merit of a large receptive field of GANs to generate good segmentation results [82].

Incorporating GAN techniques in the medical field helps enrich health care centers with various data and effectively solves data imbalance problem [87,134]. As a result, this feature facilitates solving ethical issues surrounding patients’ privacy [72], saves memory and time needed to collecting images [79], reduces costs [88], and saturates the nature of data-hungry DLMs [51].

### Recommendation

**Figure 12:**
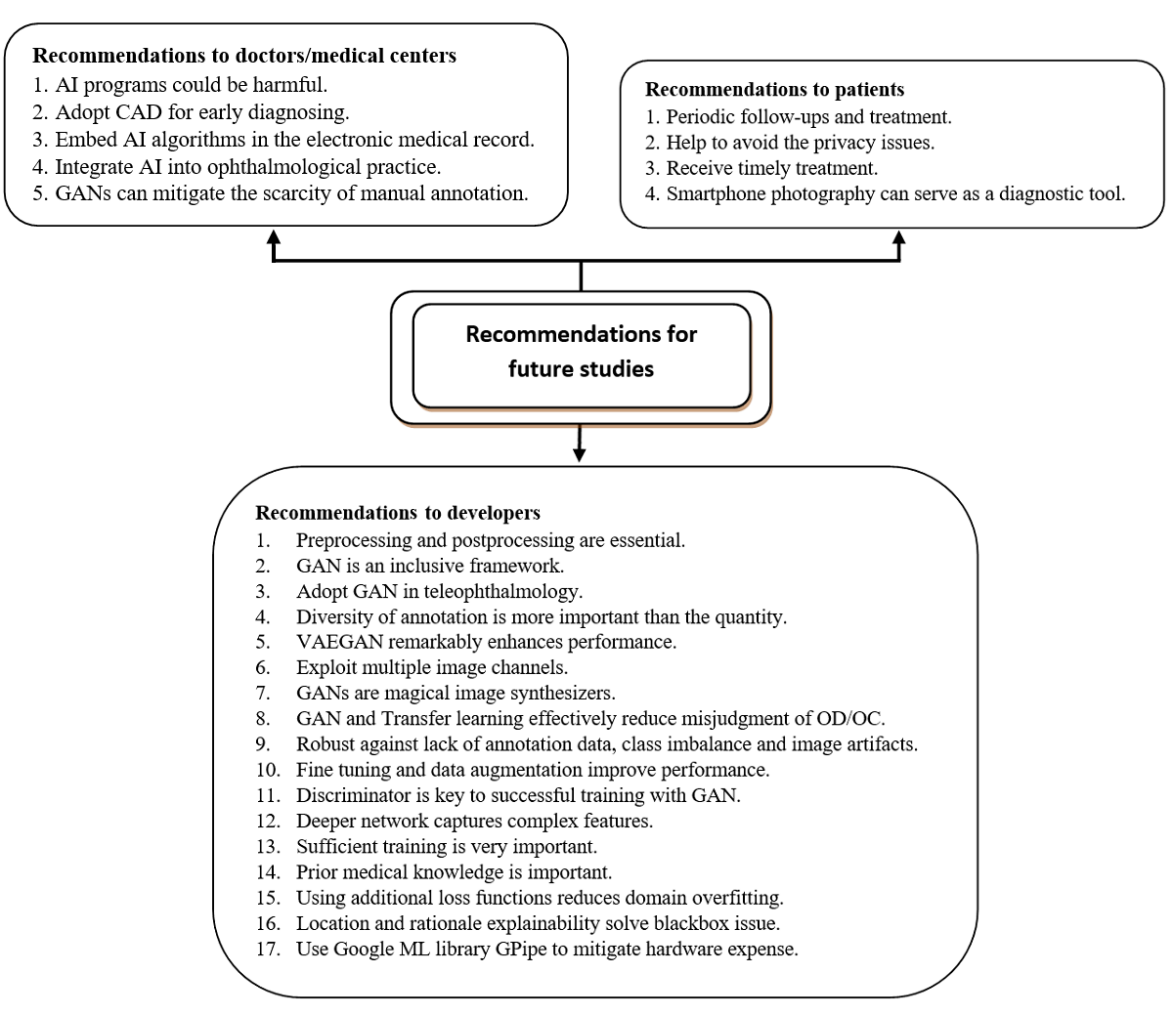
Recommendations of using GANs-based methods in glaucoma screening. AI: artificial intelligence; CAD: computed-aided design; GAN: generative adversarial network; OC: optic cup; OD: optic disc; VAEGAN: variational autoencoder with GAN.

In this section, we briefly include guidelines from the literature to alleviate existing challenges faced by researchers, doctors, medical centers, and patients, as well as present ways to achieve a correct diagnosis of retinal defects (Figure 12).

#### Recommendations to Doctors and Medical Centers

Higher-image resolutions significantly improve performance of GANs [87,148]. The key factor in obtaining GAN’s high-quality synthetic outputs is the high-resolution paired images and the architecture of the generator [46]. Moreover, annotation variety is more important than the actual number of annotations [43]. Therefore, doctors must develop a public data set with high-resolution images that meet the quality assessment system [105]. Furthermore, it must be accessible and include multiethnicities to ensure generalization capability [108]. Besides, experts must validate deep learning models on the sizable heterogeneous population under different conditions [52], as direct release of DL application without prior checking could be harmful [55].

To improve public health, reduce health care costs, and enhance patients’ perception, doctors shall adopt DL techniques in the medical field to tackle these challenges [53]. Adopting deep learning applications in magnetic resonance imaging and X-ray image processing is an interesting area of research [93]. All glaucoma studies emphasized the importance of CAD programs for early disease detection and for improvement of screening reliability [20].

In the future, GANs may be utilized to speed up AI development and application, allowing AI to comprehend and explore the environment [66]. Innovative and radical solutions for the health care system must be improved alongside glaucoma screening [106]. Significant improvements in instrumentation and interpretation can lower the cost of glaucoma screening in the future. Embedding glaucoma AI algorithms in the electronic medical record will improve outpatient management [107]. However, it is up to the physicians to lead the way in deciding how to incorporate AI in a new era of glaucoma management.

Automated retinal imaging technologies can reduce barriers to access and monitoring of the health system. Thus, AI integration into ophthalmology can improve patient care [56], help clinicians focus on patient relationships, and enhance health services [104], all of which can decrease irreversible blindness [54]. GANs can reduce the scarcity of manual data annotation and also be used as a clinical support tool [72].

#### Recommendations to Developers

A CNN in a generative learner is used for image segmentation tasks and obtaining successful outcomes [149]. GAN is an inclusive system that can be combined with various deep learning models to address problems that conventional ML algorithms cannot solve, such as poor quality of outputs, insufficient training samples, and deep feature extraction [68]. Furthermore, it outperforms conventional methods in editing and synthesizing image [69]. GAN allied with transfer learning can effectively reduce misjudgment of OD/OC in glaucoma cases and improve accuracy and generalization capability; however, better backbone network and different upsampling methods are required to improve performance [86] and exploring other downstream tasks may enhance the model’s performance [93]. Although there is a vast increase of GAN applications, further studies are required to improve its efficiency and performance [70]. Incorporating spatial information, attention-based information, feature-maps information, and image channels (RGB) to improve network performance is a current research trend [140].

GANs can generate samples with distribution close to real data. Thus, they can be used in a systematic study of parallel systems [96]. GANs or its variants remain the future trends for mitigating imbalanced learning through generating samples close to real data, or enhancing model performance when combined with VAEs [58,65,72]. Thus, it is used as a sophisticated data augmentation technique to generate heterogeneous samples and ensure prognostic characteristics of images [52].

To date, only a few studies have experienced AI technologies in teleophthalmology [56]; photography using smartphones can be used as a diagnostic tool for ocular diseases [105]. Nowadays, there is a great need for remote disease monitoring and screening [107], especially during the COVID-19 pandemic and the vast infection transmission [150]. Thus, future study should emphasize on deep learning and telemedicine/teleretinal as potential gamechangers in the eye-care field [106].

Wang et al [89] proposed a very lightweight network architecture for joint OD and OC segmentation based on the MobileNetV2 backbone, which has few parameters and half testing time compared with the XCeption backbone, which promotes the network as a mobile app for glaucoma detection. Bisneto et al [81] presented GAN and texture features for automatic detection of glaucoma, and achieved impressive results that reached up to 100% for sensitivity, specificity, and accuracy. The authors indicated a proposal to transfer their method into a mobile app in a future study.

Future research should emphasize GANs and semisupervised learning for image synthesizing, aiming to improve the classification accuracy and the quality of the generated images simultaneously [43,75,90]. Adopting GANs in the medical field remains in its infancy, with no breakthrough application yet clinically implemented for GAN-based approaches [95]. For better feature extraction, researchers must exploit full feature information on RBG channels, spatial structure, and geometry of landmarks [83]. Semantic segmentation may reduce manual labeling effort [50,74] and enhance model performance when incorporated with WGAN domain adaptation [79]. In ophthalmology diagnosis, adversarial domain adaptation can be an important and effective direction for future research [72,88,151]. In addition, exploring the relationship between the quality of the generated image and the performance of the CAD system is needed [46].

With the envision to improve deep learning performance, preprocessing and postprocessing are essential for accurate segmentation [52,80,94]. Barros et al [20] concluded that data set size has a huge impact on the results. However, Lahiri et al [43] amazingly demonstrated that annotation diversity is more important than annotation count. GAN can make use of large amounts of unlabeled data [66,87].

Regarding GAN evaluation metrics, future studies should focus on more objective and systematic evaluation methods. However, further FID examination is required [100]. Developing quantitative assessment metrics thus remains a crucial research direction [152,153]. Researchers should evaluate their segmentation performance on public data sets [74] with heterogeneous and multimodal designs using less data-hungry algorithms [105]. In addition, the performance other classifiers (eg, XGBoost) and other cGAN architectures should be examined for faster and more accurate learning [81].

For glaucoma diagnosis, CDR and ISNT metrics present substantial information to be assessed [20]. More studies are needed to assess the validity of ophthalmology applications to detect AMD, diabetic retinopathy, and glaucoma in terms of accuracy, sensitivity, and specificity [54]. AUC, sensitivity, and specificity should be included in AI studies as the bare minimum [53].

Moreover, future research may utilize fine-tuning and data augmentation techniques to effectively improve model performance [52,81,86] and increase data set size for better training, and thus, synthesizing better classifiers [77]. GANs strength lies in its discriminator [32,80]. Duplicating the generator’s structure improves robustness [94]. Adding more network layers help capture more in-depth features [82]. Training and optimizing the model remain critical [84,87], with regard to careful balancing between G and D [92]. Patch-based images should be used as input for both G and D [51,84]. U-GAN instead of U-Net should be used to improve the model’s performance [49]. Additionally, exploiting previous knowledge of vessel structure [78,80,92] is critical for accurate segmentation [91]. Objective function supported with various loss functions may enhance model performance [84]; for example, WGAN-GP can avoid gradient disappearing and enhance training [92], Dice coefficient loss function for segmenting hard images [57], and least-squares loss function with dilated convolution can enhance small vessel segmentation [42]. On top of that, topological structure loss can enhance the connectivity of A/V classification [76], whereas binary cross-entropy loss function with false-negative loss function can improve training efficiency and increase segmentation robustness [94]. Furthermore, an adversarial loss can reduce the domain overfitting [154], and Wasserstein distance is preferable for domain adaptation, as it decreases the probability of mode collapse and avoids the gradient vanishing [79]. Weight normalization along with average pooling is the best design setting when structured prediction is used with U-Net [43]. Exploring a combination of different styles instead of training dedicated models for a particular style is necessary [93]. MISH is a modern activation function that presented better results than ReLU on most current benchmark data sets [155].

To date, explainable DLMs for glaucoma screening utilizing retinal fundus images have not been proposed [156]. Researchers should focus on relational and locational explanation using saliency maps, heatmaps, or other invented methods to provide plausible explanations of DL decisions.

Lastly, future research should incorporate the distributed ML library GPipe proposed by Google [157] to mitigate hardware limitations. This may help train large-sized models and enhance performance without tuning hyperparameters [140].

#### Recommendations to Patients

Increasing the amount of data using a successful GANs synthesizer significantly saves the privacy of patients [72]. Good DLM offers timely treatment by providing wealthy information regarding patients’ eye conditions [49]. In the near future, AI can support telemedicine platforms by facilitating the self-monitoring by patients through home-based diagnosis [56]. The availability of cheap, handy smartphones may also assist as a remote diagnostic tool [105]. This eventually could improve patient’s perception and satisfactions [53], as well as encourage continuous follow-up and treatment [106].

### New Direction of DL

Recently, DLMs have achieved positive retinal disease identification and segmentation outcomes. These technologies can revolutionize our way of life, and, probably in the next few decades, the field of medicine will change rapidly [53]. However, these techniques involve expensive hardware (eg, GPU requirements) and are greedy for images by nature. Thus, more advanced data augmentation techniques must be introduced to create heterogeneous samples while preserving the prognostic features of fundus images. A possible approach in this regard is to explore GANs [52,158]. Building systematic deep learning models trained on heterogeneous and multimodal data with fewer data-hungry algorithms can boost the effectiveness of AI in clinical settings [105]. Additionally, AI algorithms should be incorporated into electronic medical records to promote outpatient management, which is another fascinating subject [107].

From the viewpoints of accessibility, cost-effectiveness, and health care protection, there is a tremendous need to promote remote glaucoma monitoring in developed countries and rural communities, allow patients with glaucoma to obtain their own IOP data with anesthesia-free and reliable tonometers [113], and enable home-based evaluation and disease control (eg, rendering home tonometry accessible at a lower cost). Most importantly, within the current situation of the COVID-19 pandemic, new directions for DLMs can be implemented via teleretinal screening apps in ophthalmic settings to maintain maximum protection for both physicians and patients at a lower cost.

Improving the quality of diagnosis in terms of class imbalance, refining the training phases of GANs, and enhancing the computation time to better diagnose glaucoma variants remain obstacles [20,52,100]. Furthermore, it is necessary to note that GANs have not been used to diagnose difficult retinal disease to date, and GAN evaluation metrics are yet another challenging path of study [68].

Finally, combining GANs with other approaches is another prospective research approach; for example, the fusion of GANs with reinforcement learning, function learning, or conventional learning to create new AI applications and facilitate the advancement of these methods is also worth investigating [66,98].

### Limitations of the Study

This most important limitation of our analysis is the number and identification of the source databases; however, the selected works form a reasonable and broadly representative selection of the chosen sources. Furthermore, the exclusion of other retinal diseases besides glaucoma, due to its severity worldwide, is considered another limitation. In addition, a quick view of the research activities on this critical retinal disease and GANs does not necessarily reflect the research community’s response.

### Conclusion

Providing adequate health services to people with retinal disorders has been a global issue. Studies are still ongoing to diagnose retinal disorders using deep learning; however, papers adopting GANs for glaucoma detection are not as abundant as those utilizing DL or ML methods. Consequently, insights into this emerging area are needed. Six papers [18,19,26-29] have worked on glaucoma classification–based GANs, and the majority tended to use GANs for segmentation or synthesizing retinal images.

The contribution of this study lies in analyzing and taxonomizing literature works in the field of glaucoma detection using GAN-based methods. To the best of our knowledge, all the previous studies generally discussed AL or DL effects on retinal diseases, and none particularly surveyed GANs for glaucoma detection. This makes our work first to address this emerging technique.

According to our taxonomy, the majority of the collected papers paid more attention to single landmark segmentation (eg, BVs) than to the segmentation of multiple landmarks. Some techniques were of tremendous or little interest (eg, the DCGAN and cGANs). Researchers worked in this field, identified their difficulties, and suggested recommendations to overcome the current and expected challenges. Other studies focused on improving GAN architectures rather than adopting them for diagnosis. To date, there has been no specific work adopting a GAN as a smartphone app or in telemedicine. Therefore, filling this gap is important for both patients and physicians to ensure fewer physical meetings during the global COVID-19 pandemic. Furthermore, new directions in this field have been explained.

## Appendix

Multimedia Appendix 1 Segmentation papers.

Multimedia Appendix 2 Segmentation and classification papers.

Multimedia Appendix 3 Classification papers.

Multimedia Appendix 4 Synthesizing images papers.

Multimedia Appendix 5 Synthesizing, segmentation, and classification paper.

Multimedia Appendix 6 Synthesizing and classification of papers.

Multimedia Appendix 7 Segmentation and synthesizing images papers.

Multimedia Appendix 8 T-shaped matrix diagram.

## Abbreviations

BV: blood vessel
CDR: cup-to-disc ratio rule
cGANs: conditional generative adversarial networks
CNN: convolutional neural network
DCGAN: deep convolutional generative adversarial network
DLM: deep learning method
DRIVE data set: digital retinal image for vessels extraction
GANs: generative adversarial networks
ISNT: inferior, superior, nasal, and temporal rule
LAG data set: large-scale-attention-based glaucoma
LSGAN: least-square GAN
OC: optic cup
OD: optic disc
ONH: optic nerve head
REFUGE data set: retinal fundus glaucoma challenge
RGB: red green blue
RL: rim loss
RNFL: retinal nerve fiber layer
ROI: region of interest
STARE data set: structured analysis of the retina
VAEGAN: variational autoencoder with GAN
WGAN: Wasserstein GAN
WGAN-GP: Wasserstein GAN-gradient penalty
