# Supplementary material for: Accuracy of Using Generative Adversarial Networks for Glaucoma Detection: Systematic Review and Bibliometric Analysis
Source: J Med Internet Res. 2021 Sep 21;23(9):e27414. doi: 10.2196/27414 (PMC8493455; doi:10.2196/27414)
Supplement: Multimedia Appendix 2 [file jmir_v23i9e27414_app2.docx]

|  | Ref | Dataset | No of images | Landmark | Sp | Se | Acc | AUC | notes |
| --- | --- | --- | --- | --- | --- | --- | --- | --- | --- |
|  |  |  |  |  |  |  |  |  |  |
| 2020 |  |  |  |  |  |  |  |  |  |
|  | 81 | Mix of DBs | 556 | OD | 100 | 100 | 100 | 1 | Dristh-GS and RIM-ONE |
