# Supplementary material for: Accuracy of Using Generative Adversarial Networks for Glaucoma Detection: Systematic Review and Bibliometric Analysis
Source: J Med Internet Res. 2021 Sep 21;23(9):e27414. doi: 10.2196/27414 (PMC8493455; doi:10.2196/27414)
Supplement: Multimedia Appendix 3 [file jmir_v23i9e27414_app3.docx]

|  | Ref | Dataset | No of images | Landmark | SE | SP | ACC | Recall | F1 Score | G-mean | notes |
| --- | --- | --- | --- | --- | --- | --- | --- | --- | --- | --- | --- |
|  |  |  |  |  |  |  |  |  |  |  |  |
| 2019 |  |  |  |  |  |  |  |  |  |  |  |
|  | 77 | ORIGA | 650 | OD+OC |  |  |  | a0.8191  b0.8737 | ^a^0.6968  ^b^0.7124 | ^a^0.8007  ^b^0.8153 | ^a^ REFUGE used in training  ^b^ ISEE used in training |
| 2020 |  |  |  |  |  |  |  |  |  |  |  |
|  | 76 | Drive | 40 | BV | 0.918 | 0.930 | 0.943 |  |  |  |  |
|  |  | private | 3119 | BV |  |  | 0.936 |  |  |  |  |
