# Supplementary material for: Accuracy of Using Generative Adversarial Networks for Glaucoma Detection: Systematic Review and Bibliometric Analysis
Source: J Med Internet Res. 2021 Sep 21;23(9):e27414. doi: 10.2196/27414 (PMC8493455; doi:10.2196/27414)
Supplement: Multimedia Appendix 4 [file jmir_v23i9e27414_app4.docx]

|  | Ref | Dataset | No of images | Landmark | AUC | ISC | SSIM | SE | SP | F1- Score | PSNR | notes |
| --- | --- | --- | --- | --- | --- | --- | --- | --- | --- | --- | --- | --- |
|  |  |  |  |  |  |  |  |  |  |  |  |  |
| 2018 |  |  |  |  |  |  |  |  |  |  |  |  |
|  | 87 | Drive | 40 | BV | ^a^0.887 ± 0.004  ^b^0.841 ± 0.009 |  |  |  |  |  |  | ^a^  trained on real images  ^b^  trained on synthetic images |
|  |  | Messidor-1 | 1200 | BV |  | ^a^0.9832 ± 0.1117  ^b^0.9671 ± 0.0307 |  |  |  |  |  |  |
|  | 58 | Mix of datasets | 2357 | BV | ^a^0.1519 ± 0.0306  ^b^0.1431 ± 0.0306 |  |  |  |  |  |  | ^a^  trained on real images  ^b^  trained on synthetic images |
|  |  |  |  | OD | ^a^0.2456 ± 0.0722  ^b^0.1776 ± 0.0339 |  |  |  |  |  |  |  |
|  |  |  |  | BG | ^a^0.6025 ± 0.0795  ^b^0.6792 ± 0.0428 |  |  |  |  |  |  |  |
|  | 93 | Drive | 40 | BV+OD |  |  | ^c^0.8980  ^d^0.8924 | 80.38 | 98.15 | 80.33 |  | ^c^  using Tub-sGAN  ^d^ using Tub-GAN  All results trained on real and synthetic images |
|  |  | Stare | 20 | BV+OD |  |  |  | 78.96 | 98.41 | 79.02 |  |  |
|  |  | HRF | 45 | BV+OD |  |  |  | 80.01 | 98.23 | 79.50 |  |  |
|  |  | NeuB1 | N/A | BV+OD |  |  |  | 87.26 | 99.54 | 85.06 |  |  |
| 2019 |  |  |  |  |  |  |  |  |  |  |  |  |
|  | 46 | Drive | 40 | BV + OD + OC |  |  | 0.9498 |  |  |  | 23.722 | Fusion with multi-channel and different landmarks |
|  |  | Drishti-GS | 101 | BV + OD + OC |  |  | ^e^0.9117 |  |  |  | ^f^25.3665 | ^e^ Using Pix2pix method with U-net MCML generator.  ^f^ Using Pix2pix method with ResNet-9 MCML (multiple-channels-multiple-landmarks) |
|  | 92 | Messidor-1 | 1200 | BV |  |  | ^g^0.61  ^h^0.73  ^i^0.81 |  |  |  |  | ^g^ Mean SSIM  ^h^ Mean of Max(SSIM)  ^i^ Maximum SSIM |
