# Supplementary material for: Accuracy of Using Generative Adversarial Networks for Glaucoma Detection: Systematic Review and Bibliometric Analysis
Source: J Med Internet Res. 2021 Sep 21;23(9):e27414. doi: 10.2196/27414 (PMC8493455; doi:10.2196/27414)
Supplement: Multimedia Appendix 5 [file jmir_v23i9e27414_app5.docx]

|  | Ref | Dataset | No of images | Landmark | SE | SP | Acc | AUC | Run time | IoU | MIoU | δ | Notes |
| --- | --- | --- | --- | --- | --- | --- | --- | --- | --- | --- | --- | --- | --- |
|  |  |  |  |  |  |  |  |  |  |  |  |  |  |
| 2019 |  |  |  |  |  |  |  |  |  |  |  |  |  |
|  | 74 | Refugee | 800 | OD  OC | 0.7 | 0.956 | 0.8278 | 0.9011 | 0.0019 | 0.8843 ± 0.0022  0.8000 ± 0.0084 | 0.8655 ± 0.0023 | N (0.0380/ 0.0008)  G (0.0401/0.0009)  C (0.0378/0.0008) | N. δ for normal  G. δ for glaucoma  C. δ for combined |
|  |  | ORIGA | 650 | OD  OC | 0.7273 | 0.8041 | 0.7657 | 0.8622 | 0.0016 | 0.9420 ± 0.0011  0.7812 ± 0.0077 | 0.8460 ± 0.0025 | N (0.0721/ 0.0024)  G (0.0649/0.0025)  C (0.0629/0.0025) |  |
