# Supplementary material for: Accuracy of Using Generative Adversarial Networks for Glaucoma Detection: Systematic Review and Bibliometric Analysis
Source: J Med Internet Res. 2021 Sep 21;23(9):e27414. doi: 10.2196/27414 (PMC8493455; doi:10.2196/27414)
Supplement: Multimedia Appendix 6 [file jmir_v23i9e27414_app6.docx]

|  | Ref | Dataset | No of images | Landmark | Synthetic Images | Sp | Se | AUC | F1-Score | notes |
| --- | --- | --- | --- | --- | --- | --- | --- | --- | --- | --- |
|  |  |  |  |  |  |  |  |  |  |  |
| 2019 |  |  |  |  |  |  |  |  |  |  |
|  | 90 | Mix of DBs | 86926 | BV | 0.2224 ± 0.0620 | 0.7986 | 0.8290 | 0.9017 | 0.8429 | One private and 14 public datasets (ORIGA-light, Drishti-GS1, RIM-ONE, sjchoi86-HRF, HRF, DRIVE, MESSIDOR, DR KAGGLE, STARE, e-ophtha, ONHSD, CHASEDB1, DRIONS-DB [23] and SASTRA |
|  |  |  |  | OD | 0.1599 ± 0.0291 |  |  |  |  |  |
|  |  |  |  | BG | 0.6177 ± 0.0555 |  |  |  |  |  |
|  | 73 | LAG | 5824 | RNFL |  |  |  | 41.2 ± 0.5 |  |  |
|  |  |  |  | RL |  |  |  | 31.8 ± 0.3 |  |  |
