# Supplementary material for: Accuracy of Using Generative Adversarial Networks for Glaucoma Detection: Systematic Review and Bibliometric Analysis
Source: J Med Internet Res. 2021 Sep 21;23(9):e27414. doi: 10.2196/27414 (PMC8493455; doi:10.2196/27414)
Supplement: Multimedia Appendix 7 [file jmir_v23i9e27414_app7.docx]

|  | Ref | Dataset | No of images | Landmark | AUC- PR | AUC-ROC | AUC | SE | SP | Dice | F1- Score | notes |
| --- | --- | --- | --- | --- | --- | --- | --- | --- | --- | --- | --- | --- |
|  |  |  |  |  |  |  |  |  |  |  |  |  |
| 2018 |  |  |  |  |  |  |  |  |  |  |  |  |
|  | 88 | Drive | 40 | BV | 0.916 | 0.984 |  |  |  | 0.832 | 0.832 |  |
|  |  | Stare | 20 | BV | 0.922 | 0.985 |  |  |  | 0.838 | 0.838 |  |
| 2020 |  |  |  |  |  |  |  |  |  |  |  |  |
|  | 51 | Drive | 40 | BV |  |  | 0.985 | 83.0 | 97.44 | 0.838 | 82.75 | using the real plus synthetic images |
