# Supplementary material for: Accuracy of Using Generative Adversarial Networks for Glaucoma Detection: Systematic Review and Bibliometric Analysis
Source: J Med Internet Res. 2021 Sep 21;23(9):e27414. doi: 10.2196/27414 (PMC8493455; doi:10.2196/27414)
Supplement: Multimedia Appendix 8 [file jmir_v23i9e27414_app8.docx]

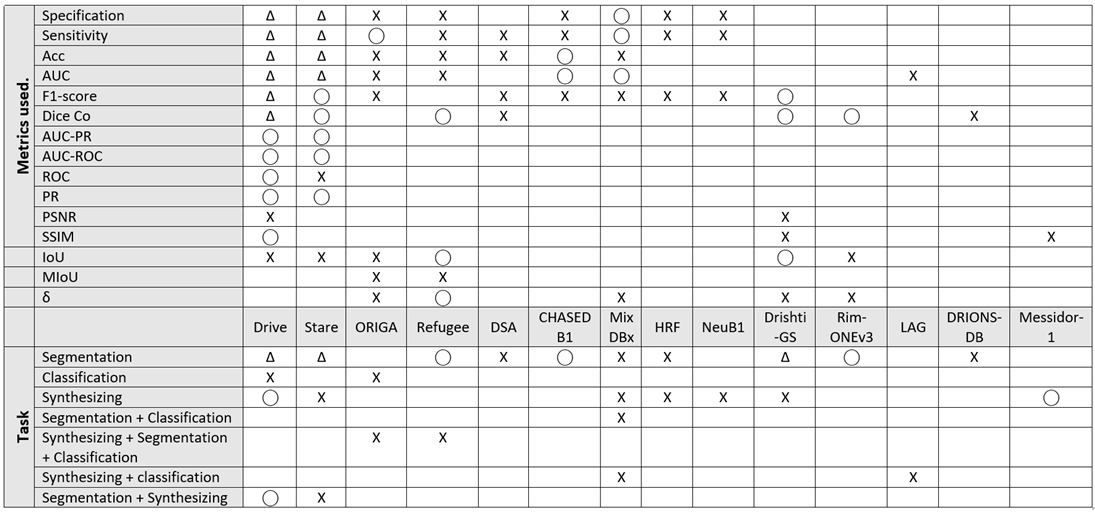


T-shaped matrix diagram shows the total use of each metric according to the dataset used. Similarly, the total use of each dataset according to a specific task. Symbol (Δ) denotes highly used (>4 time), symbol (⃝) denotes moderate use (2-4), symbol (X) denotes rarely used (= 1) and blank means unused (< 1). Note that we have ignored any metric used once by only one single dataset.
